# Cobalt: An Essential Micronutrient for Plant Growth?

**DOI:** 10.3389/fpls.2021.768523

**Published:** 2021-11-16

**Authors:** Xiu Hu, Xiangying Wei, Jie Ling, Jianjun Chen

**Affiliations:** ^1^College of Horticulture and Landscape Architecture, Zhongkai University of Agriculture and Engineering, Guangzhou, China; ^2^Institute of Oceanography, Minjiang University, Fuzhou, China; ^3^He Xiangning College of Art and Design, Zhongkai University of Agriculture and Engineering, Guangzhou, China; ^4^Department of Environmental Horticulture and Mid-Florida Research and Education Center, Institute of Food and Agricultural Sciences, University of Florida, Apopka, FL, United States

**Keywords:** cobalamin, cobalt, endophytes, essential nutrients, micronutrients, symbiosis, vitamin B_12_, transporter

## Abstract

Cobalt is a transition metal located in the fourth row of the periodic table and is a neighbor of iron and nickel. It has been considered an essential element for prokaryotes, human beings, and other mammals, but its essentiality for plants remains obscure. In this article, we proposed that cobalt (Co) is a potentially essential micronutrient of plants. Co is essential for the growth of many lower plants, such as marine algal species including diatoms, chrysophytes, and dinoflagellates, as well as for higher plants in the family *Fabaceae* or *Leguminosae*. The essentiality to leguminous plants is attributed to its role in nitrogen (N) fixation by symbiotic microbes, primarily rhizobia. Co is an integral component of cobalamin or vitamin B_12_, which is required by several enzymes involved in N_2_ fixation. In addition to symbiosis, a group of N_2_ fixing bacteria known as diazotrophs is able to situate in plant tissue as endophytes or closely associated with roots of plants including economically important crops, such as barley, corn, rice, sugarcane, and wheat. Their action in N_2_ fixation provides crops with the macronutrient of N. Co is a component of several enzymes and proteins, participating in plant metabolism. Plants may exhibit Co deficiency if there is a severe limitation in Co supply. Conversely, Co is toxic to plants at higher concentrations. High levels of Co result in pale-colored leaves, discolored veins, and the loss of leaves and can also cause iron deficiency in plants. It is anticipated that with the advance of omics, Co as a constitute of enzymes and proteins and its specific role in plant metabolism will be exclusively revealed. The confirmation of Co as an essential micronutrient will enrich our understanding of plant mineral nutrition and improve our practice in crop production.

## Introduction

Cobalt is an essential nutrient for prokaryotes, human beings, and other mammals but has not been considered an essential micronutrient for plants. Instead, this element, along with other elements, such as aluminum (Al), selenium (Se), silicon (Si), sodium (Na), and titanium (Ti), has been considered as a beneficial element for plant growth (Pilon-Smits et al., 2009; Lyu et al., 2017). An element that can improve plant health status at low concentrations but has toxic effects at high concentrations is known as a beneficial element (Pais, 1992). For an element to be considered essential, it must be required by plants to complete its life cycle, must not be replaceable by other elements, and must directly participate in plant metabolism (Arnon and Stout, 1939). It has been well-documented that there are 92 naturally occurring elements on the earth, wherein 82 of which have been found in plants (Reimann et al., 2001). Plants are able to absorb elements from soils either actively or passively due to their sessile nature. The occurrence of an element in plants, particularly in shoots, must have a purpose. Active transport of an element from roots to shoots may indicate a certain role it plays in plants. As stated in the study by Bertrand (1912), potentially, every element has a biological function that can be assessed properly against a background of a deficiency state, and every element is toxic when present at high enough concentrations, which is known as Bertrand's rule of metal necessity.

Significant progress has been made in plant mineral nutrition since the publication of Bertrand's rule (Bertrand, 1912) and the essentiality concept (Arnon and Stout, 1939). Among the beneficial elements, cobalt (Co) could potentially be an essential plant micronutrient. Co is a core element of cobalamin (vitamin B_12_ and its derivatives) and a cofactor of a wider range of enzymes and a component of different proteins in prokaryotes and animals (Maret and Vallee, 1993; Kobayashi and Shimizu, 1999; Harrop and Mascharak, 2013; Odaka and Kobayashi, 2013). Co-containing enzymes and proteins in plants require further investigation and clarification. Rhizobia and other nitrogen (N)-fixation bacteria require Co and cobalamin for fixing atmosphere dinitrogen (N_2_) into ammonia (NH_3_), providing plants with the essential macronutrient of N. Co plays a vital role in interaction with iron (Fe), nickel (Ni), and zinc (Zn) in maintaining cellular homeostasis. Similar to other essential micronutrients, plants respond to Co concentrations in soil: at low concentrations, it promotes plant growth but causes phytotoxicity at higher concentrations. However, it is different from other beneficial elements, as plants do exhibit Co deficiency when grown in soils with limited supply.

The objective of this article was to concisely review the importance of Co as a plant micronutrient including its role in N fixation, the occurrence of coenzyme or proteins, and its effects on plant growth as well as Co deficiency and toxicity. We intended that this review could raise an awareness that Co is a potentially essential micronutrient of plants, and further research is needed to confirm this proposition.

## Cobalt and Nitrogen-Fixation in Plants

Cobalt was isolated by Brandt in 1735 and recognized as a new element by Bergman in 1780 (Lindsay and Kerr, 2011). The importance of Co to living things was realized in the 1930s during the investigation of ruminant livestock nutrition in Australia (Underwood and Filmer, 1935). Co was discovered to be essential for animals as it is a component of cobalamin. Five scientists were awarded Nobel Prizes for the investigation of cobalamin (Carpenter, 2004).

### Cobalt Is a Core Element of Cobalamin

Cobalamin is a large molecule (C_63_H_88_O_14_N_14_PCo) comprised of a modified tetrapyrrole ring known as corrin with Co^3+^ in the center (Osman et al., 2021). Co is not inter-exchangeable with other metals in the cobalamin and cannot be released from the ring unless the ring is broken (Yamada, 2013), implying the significance of Co to cobalamin. There are two biologically active forms of cobalamin, namely, methylcobalamin and adenosylcobalamin in ruminants (Gonzalez-Montana et al., 2020). In human beings, Co is a cofactor of two enzymes, namely, ethylmalonyl-CoA mutase (MCM) and methionine synthase. MCM catalyzes the reversible isomerisation of l-methylmalonyl-CoA to succinyl-CoA. A deficiency of MCM causes an inherited metabolism disorder commonly known as methylmalonic aciduria. Methionine synthase utilizes cobalamin as a cofactor to produce methionine from homocysteine (Table 1). Reduced activity of this enzyme leads to megaloblastic anemia (Tjong et al., 2020). Ruminant animals produce vitamin B_12_ if there is an appropriate supply of Co in their diet. It was reported that 3 to 13% of the Co was incorporated into cobalamin by bacteria in the ruminant animals (Huwait et al., 2015).

**Table 1:** Cobalt-containing enzymes, proteins, and transporter relevant or potentially relevant to plant metabolisms.

**Type**	**Name**	**Role**	**Organism**	**References**
Corrin Co enzymes	Ethylmalonyl-CoA mutase (MCM)	Catalysis of reversible isomerisation of l-methylmalonyl-CoA to succinyl-CoA	Mammals and bacteria	Odaka and Kobayashi, 2013; Gonzalez-Montana et al., 2020
	Methionine synthase	Synthesis of methionine from homocysteine	Mammals and bacteria	Odaka and Kobayashi, 2013; Gonzalez-Montana et al., 2020
	Methylcobalamin-dependent methyltransferase	Transfer of a methyl group from different methyl donors to acceptor molecules	Mammals	Bridwell-Rabb and Drennan, 2017
	Adenosylcobalamin-dependent isomerases	Catalysis of a variety of chemically difficult 1,2-rearrangements that proceed through a mechanism involving free radical intermediates	Mammals and bacteria	Marsh and Drennan, 2001
	Ethanolamine ammonia-lyase	Conversion of ethanolamine to acetaldehyde and ammonia	*Listeria monocytogenes* and *Escherichia coli*	Harrop and Mascharak, 2013
	Ribonucleotide reductase	Catalysis of the production of deoxyribonucleotides needed for DNA synthesis	Bacteria, mammals, yeast, and plants	Elledge et al., 1992; Yoo et al., 2009
Non-corrin Co enzymes	Nitrile hydratase (NHase)	Hydration of aromatic and small aliphatic nitriles into the corresponding amides	*Rhodococcus rhodochrous* and *Pseudonocardia thermophila*	Harrop and Mascharak, 2013; Odaka and Kobayashi, 2013
	Thiocyanate hydrolase (THase)	Hydration and subsequent Hydration of thiocyanate to produce carbonyl sulfide and ammonia	*Thiobacillus thioparus* (a Gram-negative betaproteobacterium)	Harrop and Mascharak, 2013
	Methionine aminopeptidase (MA)	Cleavage of the N-terminal methionine from newly translated polypeptide chains	Bacteria, mammals, and yeast, plants	Giglione et al., 2000; Odaka and Kobayashi, 2013
	Prolidase	Cleavage of a peptide bond adjacent to a proline residue	Archaea (*Pyrococcus furiosus*), bacteria, fungi, and plants	Harrop and Mascharak, 2013; Odaka and Kobayashi, 2013
	D-xylose isomerase	Conversion of D-xylose and D-glucose into D-xylulose and D-fructose, respectively	*Streptomyces diastaticus* (an alkaliphilic and thermophillic bacterium)	Bhosale et al., 1996
	Methylmalonyl-CoA carboxytransferase	Catalysis of carboxyl transfer between two organic molecules, using two separate carboxyltransferase domains.	*Propionibacterium shermanii* (Gram-positive bacterium)	Odaka and Kobayashi, 2013
	Carbonic anhydrase or carbonate dehydratase	Catalysis of the conversion of CO_2_ to ${\text{HCO}}_{3}^{-}$ reversibly	Bacteria, fungi, algae, and plants	Jensen et al., 2020
	Carboxypeptidases	Hydrolyzation of the C-terminal residues of peptides and proteins and release free amino acids individually	Animals, bacteria, fungi, and plants	Maret and Vallee, 1993
	Urease	Catalysis of the seemingly simple hydrolysis of urea into ammonia and carbamic acid	Archaea, algae, bacteria, fungi, and plants	Carter et al., 2009
	Aldehyde decarboxylase	Decarboxylation of aldehyde	*Botryococcus braunii* (green algae)	Odaka and Kobayashi, 2013
	Bromoperoxidase	Bromination	Bacteria	Odaka and Kobayashi, 2013
Co transporters	NiCoT	Transport of Co^2+^ and Ni^2+^	*Rhodococcus rhodochrous*	Odaka and Kobayashi, 2013
	HupE/UreJ	Mediation of uptake of Ni^2+^ and Co^2+^	*Collimonas fungivorans*	Eitinger, 2013
	CbiMNQO	An energy-coupling factor (ECF) transporter for Ni^2+^ and Co^2+^	*Salmonella enterica*	Eitinger, 2013
	CorA	Transport system for Mg^2+^ and Co^2+^	Thermotoga maritima	Eitinger, 2013
	IRT1	Absorption of Fe^2+^ and Co^2+^	Plants	Korshunova et al., 1999; Conte and Walker, 2011
	FPN1	Transport of Fe^2+^ and Co^2+^ to xylem	Plants	Korshunova et al., 1999; Conte and Walker, 2011
	FPN2	Transport of Fe^2+^ and Co^2+^ to vacuole	Plants	Korshunova et al., 1999; Conte and Walker, 2011
	ARG1	An ABC transporter to transporting Ni^2+^ and Co^2+^ in chloroplast	Plants	Li et al., 2020

### Cobalamin Biosynthesis in Bacteria and Archaea

The natural forms of vitamin B_12_ are 1,5-deoxyadenosylcobalamin, hydroxycobalamin, and methylcobalamin (Nohwar et al., 2020). They are synthesized by a selected subset of bacteria and archaea (Heal et al., 2017; Guo and Chen, 2018), which include *Bacillus, Escherichia, Fervidobacterium, Kosmotoga, Lactobacillus, Mesotoga, Nitrosopumilus, Petrotoga, Propionibacterium, Proteobacteria, Pseudomonas, Rhodobacter, Rhizobium, Salmonella, Sinorhizobium, Thermosipho*, and *Thermotoga* (Doxey et al., 2015; Fang et al., 2017). Cyanocobalamin is not a natural form but commercially synthesized B_12_. The production of vitamin B_12_ by these microbes involves about 30 enzymatic steps through either aerobic or anaerobic pathways. In addition to being essential for fat and carbohydrate metabolism and synthesis of DNA, vitamin B_12_ is a cofactor of many enzymes. There are more than 20 cobalamin-dependent enzymes in those prokaryotes including diol dehydratase, ethanolamine ammonia-lyase, glutamate, and methylmalonyl-CoA mutase, methionine synthase, and ribonucleotide reductase (Marsh, 1999) (Table 1). These enzymes catalyze a series of transmethylation and rearrangement reactions (Rodionov et al., 2003). Thus, Co is essential for those archaea and bacteria.

### Cobalt Plays an Important Role in Biological Nitrogen Fixation

**Figure 1:**
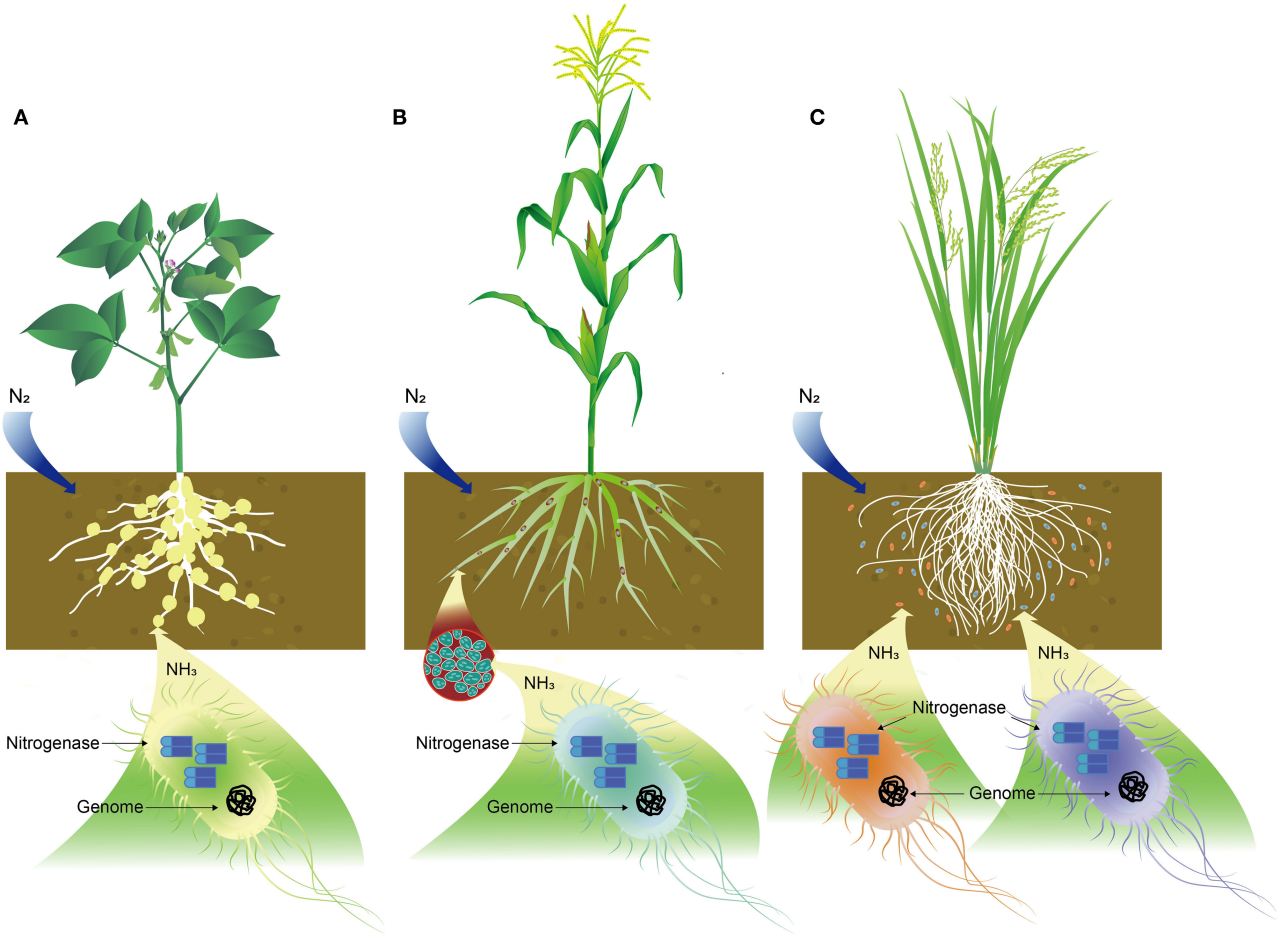
Schematic illustration of nitrogen (N) fixation in bacteria. **(A)** Symbiotic relationship of *Rhizobium* with soybean in N fixation, **(B)** endophytic bacteria, like *Azospirillum lipoferum* in corn plant root for N fixation, and **(C)** N-fixing bacteria, such as *Azotobacter* and *Azospirillum* associated with rice plant roots where cobalt or cobalamin plays important role in N fixation.

Biological N fixation is a process of converting N_2_ from the atmosphere into plant-usable form, primarily NH_3._ Biological N fixation (BNF) is carried out by a group of prokaryotes known as diazotrophs, which are listed in Table 2, including bacteria, mainly *Rhizobium, Frankia, Azotobacter, Mycobaterium, Azospirillum*, and *Bacillus*; Archaea, such as Methanococcales, Methanobacteriatles, and Methanomicrobiales, and cyanobacteria, like *Anabaena, Nostoc, Toypothrix*, and *Anabaenopsis* (Soumare et al., 2020). N_2_-fixing organisms are also classified into three categories: symbiotic, endophytic, and associated groups (Figure 1). Such classifications may not be accurate as some of them, such as those from *Acetobacter* and *Azospirillum*, could be associated, as well as endophytic bacteria.

**Table 2:** Representative nitrogen fixing bacteria.

**Type of association**	**Bacteria**	**Plants**	**References**
Symbiosis	Cyanobacteria	Bryophyte symbiosisNostoc-Gunnera symbiosisAzolla symbiosisCycad symbiosisLichen symbiosis	Adams et al., 2013
	Rhizobia (*Bradyrhizobium, Burkholderia, Ensifer*, and *Mesorhizobium*)	Legume-Rhizobia symbiosis	Andrews and Andrews, 2017
	*Frankia*	Non-legume-Frankia symbiosis: Actinorhizal plants	Wall, 2000
Endophyte	*Azospirillum amazomense*; *Bacillus* spp.; *Burkhoderia* spp.; *Gluconacetobacter diazotrophicus*; *Paenibacillus polymyxa*; and *Pseudomonas aeruginosa*	RiceMaizeRiceSugarcaneMaizeWheat	Puri et al., 2018; Rana et al., 2020
Association	*Acetobacter nitrocaptans*; *Azospirillum* spp.; *Bacillus azotofixans*; and *Pseudomonas* spp.	Sugarcane associationGrasses and cereals (maize, sorghum, wheat)Grasses, sugarcane, wheatWetland rice	Boddey and Dobereiner, 1988; Rosenblueth et al., 2018
	Cyanobacteria; *Acetobacter diazotrophicus*; *Azoarcus* spp.; *Azospirillum* spp.; and *Azotobacter* spp.	SugarcaneGrassesMaize, wheatSugarcane	Steenhoudt and Vanderleyden, 2000

#### Cobalt Is Essential for Symbiotic Bacteria in N Fixation

There are two major symbioses between N_2_-fixing bacteria and higher plants, one is rhizobia with leguminous plants and the other is *Frankia* with actinorhizal plants (Wall, 2000). The former involves more than 1,700 plant species in the family *Fabaceae*, which includes some economically important crops, such as alfalfa, beans, peas, and soybeans. More than 220 species are actinorhizal plants, which are mainly trees and shrubs forming symbiotic relationships with *Frankia*.

*Rhizobia* are gram-negative bacteria encompassing *Rhizobium, Azorhizobium, Sinorhizobium, Bradyrhizobium*, and *Mesorhizobium* (Table 2, Figure 1A). Co was identified to be essential for *Rhizobium* in the 1950s and 1960s (Ahmed and Evans, 1960; Reisenauer, 1960). *Rhizobium* uses nitrogenase to catalyze the conversion of N_2_ to NH_3_, which can be readily absorbed and assimilated by plants. Three enzymes, namely, methionine synthase, methyl malonyl-CoA mutase, and ribonucleotide reductase in *Rhizobium* and *Bradyrhizobium* species, are known to be cobalamin-dependent and significantly affect nodulation and N fixation. Early studies showed that four soybean seedlings inoculated with rhizobia supplemented with 1 μg/L Co were healthy and produced 25.3 g of dry weight. On the contrary, four rhizobia-inoculated seedlings devoid of Co encountered N-deficiency symptoms and produced 16.6 g of dry weight, a 34.4% reduction in biomass due to the absence of Co (Ahmed and Evans, 1959). A close relationship was established amongst Co supply, cobalamin content in *Rhizobium*, leghemoglobin formation, N fixation, and plant growth (Kliewer and Evans, 1963a,b). The deficiency in Co significantly affects methionine synthase by reducing methionine synthesis, which subsequently decreases protein synthesis and produces smaller-sized bacteroids (bacteria in the nodules capable of N fixation) (Marschner, 2011). Methyl malonyl-CoA mutase catalyzes the production of leghemoglobin. If Co becomes limited, leghemoglobin synthesis is directly affected, resulting in reduced N fixation and ultimately a shortage of N supply. This is because leghemoglobin can protect nitrogenase from oxygen by limiting its supply (Hopkins, 1995). Ribonucleotide reductase is a cobalamin-dependent enzyme that catalyzes the reduction of ribonucleotides to deoxyribonucleotides, which is a rate-limiting step in DNA synthesis (Kolberg et al., 2004).

The genus *Frankia* is composed of gram-positive and gram-variable actinomycetes (Wall, 2000). It infects plants through root hairs and produces nodules in the pericycle. *Frankia* in nodules develops vesicles in which nitrogenase is suited (Huss-Danell, 1997). Co is needed for the synthesis of cobalamin which is in turn needed for N fixation. Actinomyceters are known as active producers of cobalamin (Hewitt and Bond, 1966). N fixation by actinorhizal plants appears to be comparable to the magnitude as that of the legumes (Wall, 2000).

Other symbioses occur in cyanobacteria with *Gunnera* and cycads. The genus *Nostoc* infected specialized gland organs located on the stems of *Gunnera*, such as *G. chilensis* and *G. magellanica* (Johansson and Bergman, 1994). Cyanobacteria also form symbiotic relationships with cycads in a special type of root system called coralloid roots (Chang et al., 2019). It has been well-documented that cyanobacteria require Co for the biosynthesis of cobalamin (Cavet et al., 2003).

#### Cobalt and Endophytic Bacteria in N Fixation

A group of N_2_-fixing bacteria can form an endophytic relationship with many crop plants (Table 2, Figure 1B). By definition, any bacterium could be considered to be an endophytic diazotroph if (1) it can be isolated from surface-disinfected plant tissue or extracted inside the plants, (2) it proves to be located inside the plant, either intra- or inter-cellularly by *in-situ* identification, and (3) it fixes N_2_, as demonstrated by acetylene reduction and/or ^15^N-enrichment (Hartmann et al., 2000; Gupta et al., 2012). Common N_2_-fixing endophytic bacteria include *Azoarcus* spp. BH72 and *Pseudomonas stutzeri* A1501 in rice (Wang et al., 2016; Pham et al., 2017)*, Achromobacter* spp. EMC1936 in tomato (Abdel-Rahman et al., 2017), *Azospirillum lipoferum* 4B in maize (Garcia et al., 2017), *Burkholderia phytofirmans* PsJN in grape plants (Compant et al., 2008), Enterobacter cloacae ENHKU01 in pepper (Santoyo et al., 2016), *Gluconoacetobacter diazotrophicus* PaI5 in sugarcane (James et al., 2001). Other bacteria, such as *Herbaspirillum, Klebsiella*, and *Serratia* also are implicated in N_2_ fixation (Rothballer et al., 2008; Franche et al., 2009). These bacteria possess either iron or vanadium nitrogenase that fixes N_2_ into NH_3_.

The complete genome of *Azoarcus* sp. BH72 (Krause et al., 2006), *G. diazotrophicus* PAl 5 (Bertalan et al., 2009), *Herbaspirillum seropedicae* SmR1 (Pedrosa et al., 2011), and *S. marcesens* RSC-14 (Khan et al., 2017) were sequenced. Among them, genomic and proteomic profiles of *Azoarcus* sp., *Gluconoacetobacter diazotrophicus, Herbaspirillum seropedicae*, and *Serratia marcesens* have been studied (Krause et al., 2006; Gupta et al., 2012). These bacteria have co-transport systems for Co^2+^, Zn^2+^, and Cd^2+^ or Ca^2+^, Co^2+^, Zn^2+^, and Cd^2+^ as well as putative receptors for vitamin B_12_. Comparative genomic analyses of Ni, Co, and vitamin B_12_ utilization showed that both metals are widely used by the bacteria and archaea, with the most common prokaryotic transporter being Cbi/NikMNQO. Ni-Fe hydrogenase, Ni-dependent urease, B_12_-dependent ribonucleotide reductase, methionine synthase, and methymalonly-CoA mutase are the most widespread metalloproteins for Ni and Co (Zhang et al., 2009). Thus, Co is needed by these bacteria.

#### Cobalt and Plant Associated N_2_ Fixing Bacteria

Associated N_2_-fixing bacteria include *Azotobacter, Azospirillum, Beijerinckia, Burkholderia, Clostridium, Herbaspirillum, Gluconacetobacter, Methanosarcina*, and *Paenibacillus* (Table 2, Figure 1C). These bacteria are associated with the roots of a wide range of plants, including corn, rice, sugarcane, and wheat (Aasfar et al., 2021). Among them, the genus *Azotobacter* was first reported in 1901 and has been used as a biofertilizer thereafter (Gerlach and Vogel, 1902). Notable species found in soils are *A. chroococcum, A. vinelandii, A. beigerinckii, A. armeniacus, A. nigricans*, and *A. paspali* (Das, 2019). The genome of *A. vinelandii* DJ has been sequenced (Setubal et al., 2009). N fixation in these species is under aerobic conditions, and two-component proteins of Mo-dependent nitrogenase catalyze N_2_ into NH_3_. Co and vitamin B_12_ were found to be required by *A. vinelandii* OP. Additionally, 5,6-dimethylbenzimidazolylcobamide coenzyme was identified in this species, which might play an important role in N fixation (Nicholas et al., 1962). Furthermore, higher concentrations of Co were needed for *A. vinelandii* to fix N_2_ than was needed for the utilization of ammonium compounds (Evans and Kliewer, 1964). Co at a concentration of 0.1 mg/L was reported to increase N fixation in *A. chroococcum* in Jensen's medium (Iswaran and Rao Sundara, 1964). Culture of *A. chroococcum* in half-strength N-free Jensen's broth showed that N fixation was enhanced after supplemented with Co at 12.5 mg/L or 25 mg/L (Orji et al., 2018). Azotobacters were able to biosynthesize a series of vitamins, including B_12_ in chemically-defined media and dialyzed soil media (Gonzalez-Lopez et al., 1983; El-Essawy et al., 1984). In addition to *A. vinelandii* and *A. chroococcum, Pseudomonas fluorescens, Bacillus megaterium, Bacillus firmus*, and *Sinorhizobium meliloti* also produce cobalamin (Palacios et al., 2014), and the synthesized cobalamin may implicate the enhanced N fixation in these bacteria.

*Azosprillum* is another important genus of plant-associated N_2_-fixing bacteria. *A. brasilense* cultured on medium supplemented with 0.2 m*M* Co was able to accumulate Co up to 0.1 to 0.6 mg per gram of dry biomass (Kamnev et al., 2001). ^57^Co emission Mössbauer spectroscopy (EMS) studies of Co in *Azospirillum brasilense* Sp245 showed that Co activated glutamine synthetase to have two different Co forms at its active sites. *In vitro*, biochemical and spectroscopic analyses showed that Co^2+^ is among the divalent cations, along with Mg^2+^ and Mn^2+^, most effective in supporting the activity of glutamine synthetase at different adenylylation states, a key enzyme of N metabolism (Antonyuk et al., 2001).

### Nitrogen Fixing Bacteria and Crop Productivity

Nitrogen is an essential macronutrient for plants. The application of synthetic N fertilizers has greatly enhanced crop production but also has caused serious environmental problems, such as groundwater contamination and surface water eutrophication (Hansen et al., 2017). As a result, exploring the potential of BNF becomes increasingly important. The symbiotic relationship between rhizobia and legume crops was considered the most important BNF system and estimated to contribute to 227 to 300 kg N/ha/year (Roughley et al., 1995; Herridge et al., 2008). N_2_ fixation by actinorhizal plants was estimated to be 240-350 kg N/ha/year (Wall, 2000).

Nitrogen fixation by plant-associated diazotrophs has been estimated to be 60 kg N/ha/year (Gupta et al., 2006; Reed et al., 2011). Moreover, the abundance of associated diazotrophs, such as *Azotobacter* species in the soil provides not only N (Din et al., 2019) but also phosphorus and plant growth regulators, which resulted in a yield increase of up to 40% in cereals and pulse crops (Yanni and El-Fattah, 1999; Choudhury and Kennedy, 2004; Kannan and Ponmurugan, 2010; Ritika and Dey, 2014; Wani et al., 2016; Velmourougane et al., 2019). Such beneficial effects have been harnessed ecologically in the engineering of *Azotobacter* species for fixing plant needed N, while reducing the reliance on synthetic N fertilizers for crop production in an environmentally friendly manner (Wani et al., 2016; Bageshwar et al., 2017; Ke et al., 2021).

Endophytic bacteria also contribute significantly to N input. *Azoarcus* is an endophytic N_2_-fixing diazotroph, and its action in roots of kallar grass increased hay yield up to 20–40 t/ha/year without N fertilizer application in saline-sodic, alkaline soils (Hurek and Reinhold-Hurek, 2003). *Gluconoacetobacter diazotrophicus* (*Acetobacter diazotrophicus*) is the main contributor in sugarcane and can fix up to 150 kg N/ha/year (Dobereiner et al., 1993; Muthukumarasamy et al., 2005). Many C-4 energy plants, such as *Miscanthus sacchariflorus, Spartina pectinate*, and *Penisettum purpureum* can harbor endophytic bacteria, which support the N requirement of these plants (Kirchhof et al., 1997). Gupta et al. (2012) reported that N derived from the air by endophytic bacteria for rice ranged from 9.2 to 47% depending on bacterial species. These results indicate that endophytic diazotrophs have a great potential to enhance the productivity of non-leguminous crops.

The aforementioned bacteria essentially act as the same as gut bacteria in mammals by living between plant cells as endophytes, close association with roots, or symbiotically and become indispensable for plant growth and development. Microorganisms are associated with all plant organs (Wei et al., 2017), but roots have the largest number and greatest range of microbes. Thus, a plant growing under field conditions is a community, not an individual. Such associations are collectively termed “phytomicrobiome.” The phytomicrobiome is integral for plant growth and function. Microbes play important roles in plant nutrient acquisition, biotic and abiotic stress management, physiology regulation through microbe-to-plant signals, and growth regulation *via* the production of phytohormones. The foregoing discussion documents the role of Co plays in N_2_ fixing rhizosphere bacteria. If we accept that coevolution exists between microbes and plants and the phytomicrobione in general, Co should be considered as an essential element to plants as it is required by symbiotic, endophytic, and associated bacteria.

## Cobalt Coenzymes and Proteins

Cobalamin is a cofactor of adenosylcobalamin-dependent isomerases, ethanolamine ammonia-lyase, methylcobalamin-dependent methyltransferase, and ribonucleotide reductase in animals and bacteria (Table 1). Co is also a cofactor of non-corrin coenzymes or metalloproteins including aldehyde decarboxylase, bromoperoxidase-esterase, D-xylose isomerase, methionine aminopeptidase (MA), methylmalonyl-CoA carboxytransferase, nitrile hydratase (NHase), prolidase, and thiocyanate hydrolase (THase) in animals, bacteria, and yeasts. However, cobalamin-dependent enzymes or Co-proteins in plants remain obscure.

### Cobalt Proteins in Plants

There are several lines of evidence suggesting that plants may have cobalamin-dependent enzymes and Co-containing proteins: (1) The ancestor of the chloroplast is cyanobacteria (Falcón et al., 2010), and Co is required by this group of bacteria. The speculation is that Co may be needed by plants. (2) Plants have been documented to utilize cobalamin produced by symbiotic, endophytic, and associated N_2_ fixing bacteria. Cobalamin concentrations of 37, 26, and 11 μg/100 g dry weight were detected in *Hippophae rhammoides, Elymus*, and *Inula helenium*, respectively (Nakos et al., 2017). There is a possibility that cobalamin-dependent enzymes may occur in plants. Poston (1977) reported the identification of leucine 2,3-aminomutase in extracts of bean seedlings. Its activity was stimulated by coenzyme B_12_ but inhibited by unknown factors. The inhibition was removed by the addition of B_12_, suggesting the presence of a cobalamin-dependent enzyme in higher plants. Subsequently, two coenzyme B_12_-dependent enzymes: leucine 2,3-aminomutase and methylmalonyl-CoA mutase were reported in potato tubers (Poston, 1978), but methylmalonyl-CoA mutase was found to be a phosphatase (Paizs et al., 2008). (3) Co is required by lower plants, which is to be discussed in the following section. (4) Plants can take up and transport cobalamin (Mozafar, 1994; Sato et al., 2004). A recent study using fluorescent analogs to follow the uptake and transport of cobalamin showed that *Lepidium sativum* can absorb cobalamin (Lawrence et al., 2018). Seed priming with cobalamin provided significant protection against the salt stress of common beans (Keshavarz and Moghadam, 2017). The incorporation of Co in plant tissue culture media significantly improves plantlet production (Bartolo and Macey, 1989). (5) Co as a metal cofactor of some additional enzymes and proteins are briefly discussed below (Table 1).

Carbonic anhydrase or carbonate dehydratase (CA, EC: 4.2.1.1) is a metalloenzyme catalyzing the conversion of CO_2_ to ${\text{HCO}}_{3}^{-}$ reversibly in many organisms including plants, particularly C_4_ and CAM plants. Eight different CA classes have been described as α-, β-, γ-, δ-, ζ-, η-, θ-, and a recently described ι-CA in microalgae. The metalloenzymes commonly use Zn^2+^ as a metal cofactor. However, Zn^2+^ in γ class can be replaced by Co^2+^ and Fe^2+^ in prokaryotes, fungi, algae, and plants, but in δ class is only can be replaced by Co^2+^ in marine phytoplankton (Jensen et al., 2020).

Carboxypeptidases (CPSs, EC: 3.4.16–3.4.18) are proteases hydrolyzing the C-terminal residues of peptides and proteins and release free amino acids individually. CPSs are divided into serine (EC: 3.4.16), metal (EC: 3.4.17), and cysteine (EC: 3.4.18) and occur in animals, bacteria, fungi, and plants. One Zn atom is essential to the catalytic activity of native carboxypeptidase A. Zn can be removed by dialysis at low pH or with chelating agents at neutral pH, which results in the inactivation of the enzyme. The re-addition of the metal restores the dual activities of carboxypeptidase toward peptides and esters. Co was found to be more active than Zn in the enzyme toward peptides and has nearly the same activity toward esters, indicating that Co in the active site is virtually identical to that of Zn in the native enzyme (Maret and Vallee, 1993).

Methionine aminopeptidase (MAP, EC 3.4.11.18) is widely documented in animals, bacteria, yeast, and plants. It is a Co-dependent enzyme responsible for the cleavage of the N-terminal methionine from newly translated polypeptide chains. Two classes of MAPs (MAP1 and MAP2) were reported in bacteria, and at least one MAP1 and one MAP2 occur in eukaryotes (Giglione and Meinnel, 2001). In *Arabidopsis*, there are four MAP1s (MAP1A, MAP1B, MAP1C, and MAP1D) and two MAP2s (MAP2A and MAP2B), along with two class 1 peptide deformylases (PDF1A and PDF1B). The plant MAP proteins show significant similarity to the eubacterial counterparts except for MAP1A and two MAP2s. It has been documented that the substrate specificity of PDFs and both organellar and cytosolic MAPs in plants are similar to that of their bacterial counterparts (Giglione et al., 2000). The MAP from *Salmonella typhimurium* is stimulated only by Co^2+^, not by Mg^2+^, Mn^2+^, or Zn^2+^ and is inhibited by metal ion chelator EDTA. *E. coli* MAP is a monomeric protein of 29 kDa consisting of 263 residues that possess two Co^2+^ ions in its active site (Permyakov, 2021).

Prolidase (PEPD, EC 3.4.13.9) hydrolyze peptide bonds of imidodipeptides with C-terminal proline or hydroxyproline, thus liberating proline. PEPD has been identified in fungi, plants (Kubota et al., 1977), archaea, and bacteria. The preferable substrate requires metal ions Mn^2+^, Zn^2+^, or Co^2+^.

Peroxidases are isoenzymes present in all organisms, which catalyze redox reactions that cleave peroxides; specifically, it breaks down hydrogen peroxide. The study of Han et al. (2008) found that Co^2+^ at a concentration below 0.1 mM increased horseradish peroxidase activity because Co^2+^ binds with some amino acids near or in the active site of the enzyme.

Urease is an enzyme occurring in selected archaea, algae, bacteria, fungi, and plants. It catalyzes the hydrolysis of urea into ammonia and carbamic acid. The active site of urease contains two Ni^2+^ atoms that are bridged by a carbamylated lysine residue and a water molecule (Carter et al., 2009). The study of Watanabe et al. (1994) reported that urease activity of cucumber leaves was markedly reduced when Ni concentration became <100 ng/L, but supplementing Co restored urase activity. Additionally, urease was also activated by both Co and manganese (Mn) through *in vitro* assay (Carter et al., 2009).

**Figure 2:**
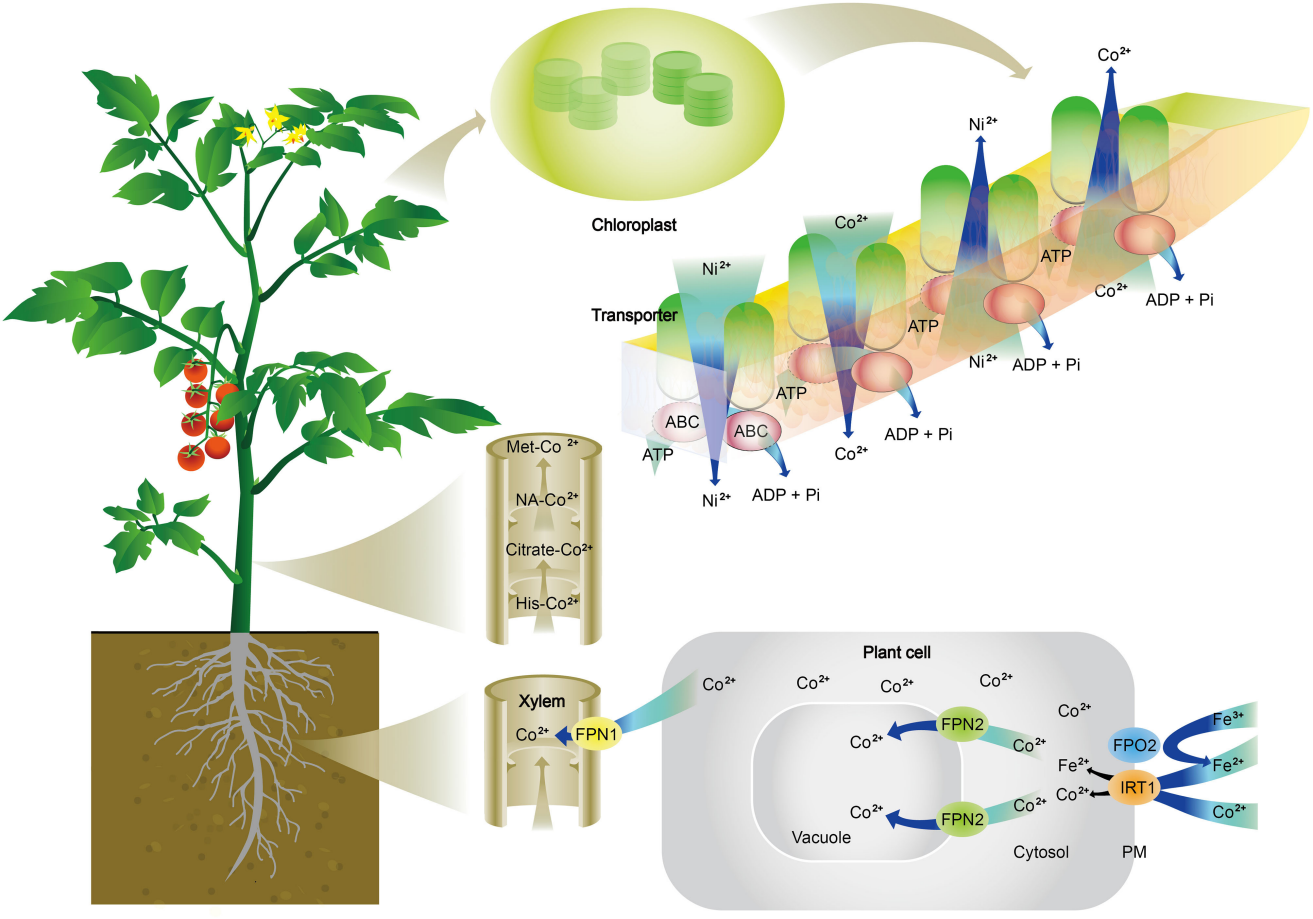
Schematic illustration of cobalt (Co^2+^) absorption, transport, and distribution in plants. Co^2+^ is absorbed from the soil into epidermal cells of roots by an iron transporter (IRT1). Once Co^2+^ is absorbed inside cells, Ferroportins (FPN1 and FPN2) are responsible for its further movement. FPN2 transports Co^2+^ into vacuoles, resulting in the sequestration of Co in root cells. FPN1 is to load Co^2+^ into the xylem. In the xylem, Co^2+^ is complexed with citrate, histidine (His), methionine (Met), or nicotianamine (NA) to be translocated to shoots. Co^2+^ is released in leaves and participate in metabolisms, which are often associated with nickel (Ni) and iron (Fe). It is shown here the Ni and Co movement in or out of chloroplasts through ATP-binding cassette (ABC) transporter in the mediation of ionic homeostasis in the chloroplast.

Cobalt transporters. Transporters specifically for Co have not been reported. The current understanding is that Co can be transported through Fe transporters (Figure 2). In *Arabidopsis thaliana*, Co is taken up from the soil into epidermal cells of roots by IRON-REGULATED TRANSPORTER 1 (IRT1), which is commonly known for absorption of Fe (Korshunova et al., 1999). Once Co is absorbed inside cells, Ferroportins, FPN1, and FPN2 are responsible for its further movement. IREG1/FPN1 is localized to the plasma membrane and expressed in the steel, indicating it is responsible for the loading of Fe to xylem, and FPN2 is situated the in vacuolar membrane and involved in buffering Fe concentration in the cytosol (Morrissey et al., 2009). Truncated *FPN2* causes an elevated level of Co in shoots, while the loss of *FPN1* abolishes Co accumulation in shoots. A double mutant of *fpn1 fpn2* is unable to sequester Co in root vacuole and cannot transport Co to shoots. These results suggest that Co is likely absorbed and transported in the same way as Fe in plants (Figure 2). Additionally, an ATP-binding cassette (ABC) transporter from *Arabidopsis* has also been reported to transport Co, Ni, and Pb (Morel et al., 2009). Co movement in leaves is also associated with Ni, and Ni and Co movement in or out of chloroplasts are through an ABC transporter in the mediation of ionic homeostasis in the chloroplast of rice (Li et al., 2020).

### Cobalt Substitution of Other Metals

A characteristic of Co is its ability to substitute for other transition metals in a large number of enzymes. Maret and Vallee (1993) listed 37 Co-substituted metalloproteins, of which 24 are native to Zn, nine to copper (Cu), and four to Fe. These enzymes mainly occur in animals, bacteria, and yeast, while a few are in plants. Such a characteristic is closely related to the properties of Co with other metals. The ionic radius of Co^2+^ is 0.76 Å, which is similar to 0.74 Å of Zn^2+^, 0.69 Å for Cu^2+^, and 0.76 Å for Fe^2+^. Additionally, based on the available Protein Data Bank structures with Co^2+^, the study Khrustalev et al. (2019) found that Co^2+^ is commonly bound by cation traps. The traps are formed by relatively negatively charged regions of random coil between a β stand and α helix and between two β strands in which His, Asp, and Glu residues are situated. On the other hand, these sites are also occupied by other metals ions, such as Cu^2+^, Mg^2+^, Mn^2+^, and Zn^2+^, which play significant roles as catalysts. As a result, Co^2+^ could rather readily substitute for these ions in the active sites of enzymes. Additionally, based on the FIND-SITE-metal, a program for the prediction of the metal-binding site, the study of Brylinski and Skolnick (2011) found that Zn, due to a lower coordination number preference, is typically chelated with Cys and His, and His residues have a strong preference for Co, Cu, Fe, Ni, and Zn atoms. Thus, Co is able to replace Cu, Fe, Ni, and Zn in the active sites of enzymes. For example, Co addition alleviated Zn limitation in production of *Thalassiosira weissflogii*, which was due to Co substitution of Zn in the main isoform of carbonic anhydrase (Yee and Morel, 1996). Co substitution of Zn was also reported in two northeast Pacific isolates of diatoms *Pseudo-nitzschia delicatissima* UNC1205 and *Thalassiosira* spp. UNC1203 (Kellogg et al., 2020). Co^2+^ has been used as a spectroscopically active substitute for Zn^2+^ in enzymes (Bennett, 2010). Substitution of tetrahedral Zn^2+^ by higher-coordinate Co^2+^ often results in a catalytically active species, sometimes with catalytic properties perhaps unexpectedly similar to those of the native enzyme. In the vast majority of cases, no other transition ion than Co^2+^ provides a better substitute for Zn^2+^ (Maret and Vallee, 1993; Bennett, 2010). Due to these reasons, Co specific enzymes or proteins have not been conclusively identified. With the advance of omics, functions of a large number of gene sequences have not been assigned. Using the FIND-SITE-metal, a program developed for prediction of the metal-binding site, Brylinski and Skolnick (2011) predicted that about 10,953 putative metal-binding proteins in human proteome were bound with Ca, 10,534 bound with Mg, 8,681 with Zn, 1,863 with Fe, 1,246 with Mn, 652 with Co, 476 with Cu, and 403 with Ni. The predicted binding proteins with Co are greater than Cu and Ni in humans. Based on this assignment in the human proteome, it could be extremely difficult to believe that there are no Co-containing enzymes and proteins in plants.

## Cobalt Is Essential for Lower Plants

Lower plants are commonly known as non-vascular plants because they do not have xylem and phloem vascular systems. Non-vascular plants are generally divided into bryophytes and algae.

### Bryophytes

Bryophytes are seedless plants including Anthocerotophyta (hornworts), Bryophyta (mosses), and Marchantiophyta (liverworts) (Davies et al., 2020). This group of plants is able to absorb Co from air, soil, and water. In an early geochemical survey performed in Wisconsin and adjacent states and Missouri and Kentucky in the US, the study of Shacklette (1965) documented that the mean concentration of Co in 38 samples of liverworts and mosses was 32 mg/kg, and the concentration in the lower plants was closely related to the amount of the element in the soil, suggesting they act as a bioindicator of Co concentration in the environment (Baker, 1981). Mosses sampled from streams of the Idaho Cobalt Belt (U.S.) showed that Co concentrations in the plants almost perfectly correlated with those in the sediments, and the maximum content of Co (2,000 mg/kg) in moss ash corresponded to the maximum concentration of 320 mg/kg in the sediment (Erdman and Modreski, 1984). Mosses, such as *Bryum argenteum* and *Hypnum cupressiforme* were also considered to be bioindicators for monitoring heavy metal contamination in the air (Andić et al., 2015). Interestingly, the accumulation of Co did not cause any physiological damages to plants, but their growth was further enhanced.

The ability to take up Co could be related to the non-vascular nature and unidentified transporter. A radiolabel study showed that the total amount of ^60^Co accumulated in *P. commune* and *D. scoparium* under given conditions were 7.1 and 6.1 mg/kg, respectively. More than 95% of ^60^Co in *D. scoparium* was localized extracellular, while 70% of ^60^Co in *P. commune* was localized extracellular and about 20% localized intracellularly. These results showed that Co was largely adsorbed extracellularly, and there were unidentified transporters regulating the transport of Co into intracellular sites.

The enhanced growth could be in part attributed to the symbiotic relationship with cyanobacteria. Some bryophytes, primarily liverworts, and hornworts can form a symbiosis with cyanobacteria, such as *Nostoc* spp. After infection, *Nostoc* underwent some morphological and physiological changes by reducing growth rate and CO_2_ fixation but enhancing the fixation of N_2_ as well as releasing fixed N compounds to the plants. Cyanobacteria, like rhizobia, require cobalamin as a cofactor for nitrogenase complex to fix N_2_ (Böhme, 1998). Thus, cyanobacteria-bryophyte symbioses require Co.

### Algae

Algae constitute a polyphyletic group ranging from unicellular microalgae, like chlorella and diatoms to multicellular forms, such as the giant kelp, seaweeds, and charophytes (Barsanti and Gualtieri, 2006). Co is essential to some marine algal species, including charophyte, diatoms, and dinoflagellates (Nagpal, 2004). Green alga *Chlorella salina* exhibited two phases of uptake of Co^2+^ (Garnham et al., 1992). The initial phase was rapid and independent of metabolism, and the second phase was slow and dependent on metabolism. Competition studies showed that the Co^2+^ uptake system was different from that for Mg^2+^, Mn^2+^, and Zn^2+^. The greatest amount of Co was associated with the cell wall. Co concentrations in the cytosol were 0.17 mM but 2.89 mM in the vacuole, suggesting that Co transport was well-controlled in *C. salina*. In the work of Czerpak et al. (1994), they studied the responses of a freshwater green alga *Chlorella pyrenoidosa* to different levels of Co and found that Co in a range from 5 to 50 mM significantly enhanced the growth of *Chlorella pyrenoidosa*, including 150–160 and 50–60% increase in fresh and dry weights, respectively. Such increase was related to the increase of chlorophylls *a* and *b* by 45–65%, water-soluble proteins by 19–20%, total carotenoids 55–65%, and monosaccharides content 55–60%, when compared with the culture devoid of Co. Although mechanisms behind the stimulating effects have not been elucidated, it is likely due to the biosynthesis of cobalamin that enhanced alga growth. Two cobalamin coenzyme 5′-deoxyadenosylcobalamin and methylcobalamin occurred in green alga *C. vulgaris*, and the addition of cobalamin significantly stimulated green alga growth (Watanabe et al., 1997). Moreover, *C. vulgaris* grown in Bold's basal medium supplemented with 2 and 2.5 μM CoCl_2_ produced 166.23 and 173.32 μg vitamin B_12_ per 100 g dry weight (Jalilian et al., 2019). Additionally, many algal species require different combinations of cobalamin, vitamin B_1_, and B_7_ (Croft et al., 2005) as they do not have pathways to synthesize cobalamin or may use alternative cobalamin-independent routes bypassing the need for the vitamin (Cruz-Lopez and Maske, 2016; Yao et al., 2018). As Co is a constituent of cobalamin, Co is required by those algae.

Some algal species, such as those in the genera *Coccomyxa* and *Elliptochloris* as well as diatoms form symbiotic relationships with cyanobacteria (Grube et al., 2017). Co is required for the growth of cyanobacteria, such as *Anabaenza cylindrica* Lemm (Holm-Hansen et al., 1954) and *Prochlorococcus* (Hawco et al., 2020) as they need it for N fixation in specialized cells called heterocysts. Thus, algal species symbiotic with cyanobacteria require Co for N-fixation.

## Cobalt Improves the Growth of Higher Plants

Cobalt content in the crust of the earth ranges from 15 to 30 mg/kg (Roberts and Gunn, 2014). Co in soils is closely related to the weathering of parental minerals, such as cobaltite, smaltite, and erythrite (Bakkaus et al., 2005) as well as Co pollution (Mahey et al., 2020). Co in the surface soils of the world varies from 4.5 to 12 mg/kg with the highest level occurring in heavy loamy soils and the lowest in organic and light sandy soils (Kabata-Pendias and Mukherjee, 2007). However, Co in reference soil samples was found to differ from 5.5 to 29.9 mg/kg in the United States (U.S.) and 5.5 to 97 mg/kg in Chinese soils (Govindaraju, 1994). Pilon-Smits et al. (2009) suggested that soil Co concentrations generally range from 15 to 25 mg/kg.

### Cobalt in Higher Plants

Plants absorb Co. Table 3 lists Co concentrations in over 140 non-hyperaccumulating species ranging from 0.04 to 274 mg/kg. Average concentrations of Co in grasses vary from 60 to 270 μg/kg and in clover differ from 100 to 570 μg/kg across Australia, Finland, Germany, Great Britain, Japan, New Zealand, Poland, Sweden, and the US (Kabata-Pendias and Mukherjee, 2007). Legumes absorb more Co than grasses. Plants that accumulate metals to a level 100-fold higher than those typically recorded in common plants are known as hyperaccumulators (Brooks, 1998).

**Table 3:** The concentration of cobalt in higher plants with the exclusion of cobalt hyperaccumulators.

**Family**	**Species**	**Common name**	**Plant organ**	**Content (mg/kg)**	**References**
Acanthaceae	*Lophostachys villosa* Pohl	Lophostachys	Leaves	31.00	Van der Ent and Reeves, 2015
Adiantaceae	*Taenitis blechnoides* (Willd.) Sw.	Ribbon fern	Leaves	22.00	Van der Ent and Reeves, 2015
Amaranthaceae	*Aerva lanata* (L.) Juss.	Gorakhdi	Whole plants	12.70	Rajakaruna and Bohm, 2002
Amaranthaceae	*Pfaffia sarcophylla* Pedersen	Pfaffia	Leaves	13.00	Van der Ent and Reeves, 2015
Anacardiaceae	*Gluta wallichii* (Hook.f.) Ding Hou	Gluta	Leaves	5.00	Van der Ent and Reeves, 2015
Anisophylleaceae	*Anisophyllea disticha* (Jack) Baill.	Mousedeer plant	Leaves	4.00	Van der Ent and Reeves, 2015
Apocynaceae	*Calotropis gigantea* L.R.Br.	Yercum fiber	Whole plants	0.84	Rajakaruna and Bohm, 2002
Apocynaceae	*Carissa spinarum* L.	Bush plum	Stems, leaves and flowers	1.60	Rajakaruna and Bohm, 2002
Arecaceae	*Phoenix farinifera* Roxb.	Ceylon date palm	Whole plants	0.04	Rajakaruna and Bohm, 2002
Aristolochiaceae	*Thottea triserialis* Ding Hou	Thottea	Leaves	5.00	Van der Ent and Reeves, 2015
Asparagaceae	*Asparagus zeylanicus* Hook.f.	Asparagus	Whole plants	0.90	Rajakaruna and Bohm, 2002
Asteraceae	*Anthemis cretica* L.	Mountain dog-daisy specie	Shoots	8.90	Koleli et al., 2015
Asteraceae	*Eupatorium odoratum* L.	Siam weed	Whole plants	0.50–3.10	Rajakaruna and Bohm, 2002
Asteraceae	*Blumea balsamifera* (L.) DC.	Bukadkad	Leaves	2.00	Van der Ent and Reeves, 2015
Asteraceae	*Vernonia holosericea* Mart. ex DC.	Ironweed	Leaves	21.00	Van der Ent and Reeves, 2015
Berberidaceae	*Podophyllum peltatum* L.	May-apple	Shoots	0.60	Koleli et al., 2015
Betulaceae	*Betula pubescens* Ehrh.	Birch	Leaves	0.36	Reimann et al., 2001
Bignoniaceae	*Zeyheria digitalis* (Vell.) Hoehne and Kuhlm	Zeyheria	Leaves	2.00	Van der Ent and Reeves, 2015
Blechnaceae	*Blechnum borneense* C.Chr.	Hard fern	Leaves	10.00	Van der Ent and Reeves, 2015
Boraginaceae	*Anchusa granatensis* Boiss.	Anchusa	Shoots	0.90	Koleli et al., 2015
Boraginaceae	*Onosma bracteosum* Hausskn. and Bornm.	Onosma	Shoots	6.60	Koleli et al., 2015
Brassicaceae	*Alyssum minus* (L.) Rothm.	Wild Alyssum	Shoots	1.20	Koleli et al., 2015
Brassicaceae	*Alyssum murale* Waldst. and Kit.	Yellowtuft	Shoots	7.70	Koleli et al., 2015
Brassicaceae	*Aurinia saxatilis* (L.) Desv.	Basket of gold	Roots	37.00	Homer, 1991
Brassicaceae	*Aurinia saxatilis* (L.) Desv.	Basket of gold	Leaves	117.00	Homer, 1991
Brassicaceae	*Brassica juncea* (L.) Czern	Brown-mustard	Stems, leaves, and flowers	25.50	Malik et al., 2000
Brassicaceae	*Thlaspi elegans* Boiss.	Thlaspi	Shoots	6.40	Koleli et al., 2015
Campanulaceae	*Campanula rapunculoides* L.	Creeping bellflower	Shoots	0.70	Koleli et al., 2015
Caryophyllaceae	*Dianthus arpadianus* Ade and Born.	Dianthus	Shoots	0.30	Koleli et al., 2015
Caryophyllaceae	*Silene burchelli* var. *angustifolia* Sond.	Gunpowder plant	Shoots	250.00	Baker et al., 1983
Chrysobalanaceae	*Parinari elmeri* Merri.	Parinari	Leaves	138.00	Van der Ent and Reeves, 2015
Clusiaceae	*Mesua paniculate* (L.) Jack	Chinese box	Leaves	77.00	Van der Ent and Reeves, 2015
Compositae	*Epaltes divaricate* (L.) Cass.	Narrow-Leaf epaltes	Whole plants	15.60	Rajakaruna and Bohm, 2002
Convolvulaceae	*Evolvulus alsinoides* L.	Little glory	Whole plants	17.10	Rajakaruna and Bohm, 2002
Convolvulaceae	*Jacquemontia* sp.	Clustervine	Leaves	16.00	Van der Ent and Reeves, 2015
Cornaceae	*Nyssa aquatica* L.	Water tupelo	Leaves	156.00	McLeod and Ciravolo, 2007
Cornaceae	*Nyssa aquatica* L.	Water tupelo	Leaves	24.50	Wallace et al., 1982
Cornaceae	*Nyssa sylvatica* Marsh.	Black gum	Mature foliage	27.20	Thomas, 1975
Cornaceae	*Nyssa sylvatica* var. *biflora* (Walt.) Sarg.	Black gum	Leaves	267.00	McLeod and Ciravolo, 2007
Cyperaceae	*Fimbristylis falcata* (Vahl) Kunth	Fimbristylis	Whole plants	16.30	Rajakaruna and Bohm, 2002
Dennstaedtiaceae	*Lindsaea gueriniana* (Gaudich.) Desv.	Goldenbush	Leaves	5.00	Van der Ent and Reeves, 2015
Dennstaedtiaceae	*Tapeinidium acuminatum* K.U. Kramer	Tapeinidium ferns	Leaves	22.00	Van der Ent and Reeves, 2015
Droseraceae	*Drosera montana* A.St.-Hil.	Sundews	Leaves	34.00	Van der Ent and Reeves, 2015
Ebenaceae	*Diospyros lanceifolia* Roxb.	Common Malayan ebony	Leaves	2.00	Van der Ent and Reeves, 2015
Equisetaceae	*Equisetum arvense* L.	Bottlebrush	Shoots	0.80	Koleli et al., 2015
Ericaceae	*Empetrum nigrum* L.	Crow-berry	Leaves	0.05	Reimann et al., 2001
Ericaceae	*Vaccinium myrtillus* L.	Blue-berry	Leaves	0.04	Reimann et al., 2001
Ericaceae	*Vaccinium vitis-idaea* L.	Cow-berry	Leaves	0.04	Reimann et al., 2001
*Euphorbiaceae*	*Croton bonplandianus* Baill.	Bonpland's croton	Stems, leaves and flowers	1.60	Rajakaruna and Bohm, 2002
Euphorbiaceae	*Croton griffithii* Hook.f.	Griffith's spurge	Leaves	10.00	Van der Ent and Reeves, 2015
Euphorbiaceae	*Drypetes caesia* Airy Shaw	Drypetes	Leaves	2.00	Van der Ent and Reeves, 2015
Euphorbiaceae	*Euphorbia macrostegia* Boiss.	Persian wood spurge	Shoots	0.90	Koleli et al., 2015
Euphorbiaceae	*Euphorbia rubicunda* Blume	Chicken weed	Stems, leaves and flowers	1.30	Rajakaruna and Bohm, 2002
Euphorbiaceae	*Euphorbia selloi* (Klotzsch and Garcke) Boiss.	Spurge	Leaves	72.00	Van der Ent and Reeves, 2015
Euphorbiaceae	*Phyllanthus* sp.	Leaf-flower	Leaves	85.00	Van der Ent and Reeves, 2015
Fabaceae	*Mimosa pudica* L.	Mimosa plant	Leaves	0.04	Van Tran and Teherani, 1989
Fabaceae	*Dalbergia beccarii* Prain	Beccari's dalbergia	Leaves	4.00	Van der Ent and Reeves, 2015
Iridaceae	*Gladiolus italicus* Miller	Field gladiolus	Shoots	1.50	Koleli et al., 2015
Iridaceae	*Sisyrinchium luzula* Klotzsch ex Klatt	Blue-eyed grass	Leaves	11.00	Van der Ent and Reeves, 2015
Labiatae	*Mentha piperita* L.	Mint	Shoots	0.04–0.17	Ciotea et al., 2021
Labiatae	*Ocimum basilicum* L.	Basil	Shoots	0.11–0.16	Ciotea et al., 2021
Labiatae	*Rosmarinus officinalis* L.	Rosemary	Shoots	0.07- 0.14	Ciotea et al., 2021
Lamiaceae	*Clerodendrum infortunatum* L.	Hill glory bower	Stems, leaves and flowers	0.60	Rajakaruna and Bohm, 2002
Lamiaceae	*Crotalaria biflora* L.	Two-flower rattlebox	Stems, leaves and flowers	15.90	Rajakaruna and Bohm, 2002
Lamiaceae	*Geniosporum tenuiflorum* (L.) Merr.	Holy basil	Whole plants	10.80	Rajakaruna and Bohm, 2002
Lamiaceae	*Leucas zeylanica* (L.) R.Br.	Ceylon leucas	Whole plants	3.30	Rajakaruna and Bohm, 2002
Lamiaceae	*Leucas zeylanica* (L.) R.Br.	Ceylon leucas	Whole plants	9.40	Rajakaruna and Bohm, 2002
Lamiaceae	*Ajuga reptans* L.	Bugleweed	Shoots	0.90	Koleli et al., 2015
Lamiaceae	*Haumaniastrum katangense* (S. Moore) Duvign. Plancke	Copper flower	Leaves	260.00	Morrison, 1979
Lamiaceae	*Sideritis trojana* Bornm.	Sideritis	Shoots	0.90	Koleli et al., 2015
Lamiaceae	*Thymus pulvinatus* Celak	Common thyme	Shoots	0.20	Koleli et al., 2015
Lamiaceae	*Hypenia macrantha* (A.St.-Hil. ex Benth.) Harley	Hypenia	Leaves	10.00	Van der Ent and Reeves, 2015
Lamiaceae	*Lippia* aff. *geminata*	Lippia	Leaves	11.00	Van der Ent and Reeves, 2015
Lamiaceae	*Lippia* sp.	Lippia	Leaves	14.00	Van der Ent and Reeves, 2015
Leguminoseae	*Tephrosia purpurea* (L.) Pers.	Wild indigo	Stems, leaves and flowers	5.20	Rajakaruna and Bohm, 2002
Leguminoseae	*Baptisia australis* (L.) R. Br. ex Ait. f.	Blue false indigo	Shoots	0.50	Koleli et al., 2015
Leguminoseae	*Vicia cassubica* L.	Vicia	Shoots	5.50	Koleli et al., 2015
Liliaceae	*Allium cepa* L.	Onion	Shoots	3.50	Koleli et al., 2015
Liliaceae	*Asphodelus aestivus* Brot.	Summer asphodel	Shoots	0.80	Koleli et al., 2015
Loganiaceae	*Norrisia* sp. 1	Norrisia	Leaves	8.00	Van der Ent and Reeves, 2015
Malvaceae	*Abutilon indicum* (L.) Sweet	Abutilon	Stems, leaves and flowers	0.80	Rajakaruna and Bohm, 2002
Malvaceae	*Hibiscus rhodanthus* Gürke ex Schinz	Dwarf red hibiscus	Leaves	21.00–1,971.00	Faucon et al., 2007
Malvaceae	*Sida acuta* Burm.f	Wire weed	Whole plants	0.30	Rajakaruna and Bohm, 2002
Malvaceae	*Waltheria indica* L.	Sleepy morning	Whole plants	1.33	Rajakaruna and Bohm, 2002
Melastomataceae	*Pterolepis* sp. nov.	Pterolepis	Leaves	11.00	Van der Ent and Reeves, 2015
Myrtaceae	*Syzygium* cf. *pterophera*	Syzygium	Leaves	7.00	Van der Ent and Reeves, 2015
Myrtaceae	*Syzygium clavatum* (Korth.) Merr. and L.M.Perry	Syzygium	Leaves	3.00	Van der Ent and Reeves, 2015
Phyllanthaceae	*Actephila* sp. nov.	Actephila	Leaves	65.00	Van der Ent et al., 2015
Phyllanthaceae	*Antidesma coriaceum* Tul.	Antidesma	Leaves	2.00	Van der Ent et al., 2015
Phyllanthaceae	*Aporosa benthamiana* Hook.f.	Aporosa	Leaves	6.00	Van der Ent et al., 2015
Phyllanthaceae	*Aporosa falcifera* Hook.f.	Aporosa	Leaves	18.00	Van der Ent et al., 2015
Phyllanthaceae	*Aporosa lucida (*Miq.) Airy Shaw	Aporosa	Leaves	18.00	Van der Ent et al., 2015
Phyllanthaceae	*Baccaurea lanceolata (*Miq.) Müll.Arg.	Baccaurea	Leaves	179.00	Van der Ent et al., 2015
Phyllanthaceae	*Breynia coronata* Hook.f.	Breynia	Leaves	4.00	Van der Ent et al., 2015
Phyllanthaceae	*Cleistanthus ellipticus* Hook.f.	Cleistanthus	Leaves	6.00	Van der Ent et al., 2015
Phyllanthaceae	*Cleistanthus gracilis* Hook.f.	Cleistanthus	Leaves	10	Van der Ent et al., 2015
Phyllanthaceae	*Cleistanthus myrianthus* (Hassk.) Kurz	Cleistanthus	Leaves	2.00	Van der Ent et al., 2015
Phyllanthaceae	*Cleistanthus gracilis* Hook.f.	Cleistanthus	Leaves	189.00	Van der Ent and Reeves, 2015
Phyllanthaceae	*Glochidion angulatum* C.B.Rob.	Glochidion	Leaves	23.00	Van der Ent et al., 2015
Phyllanthaceae	*Glochidion arborescens* Blume	Glochidion	Leaves	272.00	Van der Ent et al., 2015
Phyllanthaceae	*Glochidion borneense* (Müll.Arg.) Boerl.	Glochidion	Leaves	21.00	Van der Ent et al., 2015
Phyllanthaceae	*Glochidion brunneum* Hook.f.	Glochidion	Leaves	38.00	Van der Ent et al., 2015
Phyllanthaceae	*Glochidion calospermum* Airy Shaw	Glochidion	Leaves	13.00	Van der Ent et al., 2015
Phyllanthaceae	*Glochidion* cf. *lanceisepalum*	Glochidion	Leaves	9.00	Van der Ent and Reeves, 2015
Phyllanthaceae	*Glochidion lanceilimbum* Merr.	Glochidion	Leaves	13.00	Van der Ent et al., 2015
Phyllanthaceae	*Glochidion littorale* Blume	Glochidion	Leaves	8.00	Van der Ent et al., 2015
Phyllanthaceae	*Glochidi on lutescens* Blume	Glochidion	Leaves	2.00	Van der Ent et al., 2015
Phyllanthaceae	*Glochidion mindorense* C.B.Rob.	Glochidion	Leaves	16.00	Van der Ent and Reeves, 2015
Phyllanthaceae	*Glochidion monostylum* Airy Shaw	Glochidion	Leaves	5.00	Van der Ent et al., 2015
Phyllanthaceae	*Glochidion rubrum* Blume	Glochidion	Leaves	14.00	Van der Ent et al., 2015
Phyllanthaceae	*Glochidion rubrum* Blume	Glochidion	Leaves	25.00	Van der Ent and Reeves, 2015
Phyllanthaceae	*Glochidion singaporense* Gage	Glochidion	Leaves	120.00	Van der Ent et al., 2015
Phyllanthaceae	*Glochidionobscurum* (Roxb. ex Willd.) Blume	Glochidion	Leaves	8.00	Van der Ent et al., 2015
Phyllanthaceae	*Glochidionsuperbum* Baill. ex Müll.Arg.	Great-leafed pin-flower Tree	Leaves	22.00	Van der Ent et al., 2015
Phyllanthaceae	*Phyllanthus amarus* Schumach. and Thonn.	Sleeping plan	Leaves	38.00	Van der Ent et al., 2015
Phyllanthaceae	*Phyllanthus balgooyi* Petra Hoffm. and A.J.M.Baker	Phyllanthus	Leaves	26.00	Van der Ent et al., 2015
Phyllanthaceae	*Phyllanthus balgooyi* Petra Hoffm. and A.J.M.Baker	Phyllanthus	Leaves	11.00	Van der Ent and Reeves, 2015
Phyllanthaceae	*Phyllanthus kinabaluicus* Airy Shaw	Phyllanthus	Leaves	109.00	Van der Ent et al., 2015
Phyllanthaceae	*Phyllanthus lamprophyllus* Müll.Arg.	Phyllanthus	Leaves	11.00	Van der Ent et al., 2015
Phyllanthaceae	*Phyllanthus myrtifolius* (Wight) Müll.Arg.	Mousetail plant	Stems, leaves and flowers	0.50	Van der Ent et al., 2015
Phyllanthaceae	*Phyllanthus pulcher* Wall. ex Müll.Arg.	Tropical leaf-flower	Leaves	31.00	Van der Ent et al., 2015
Phyllanthaceae	*Phyllanthus reticulatus* Poir.	Black-honey shrub	Leaves	5.00	Van der Ent et al., 2015
Phyllanthaceae	*Phyllanthus* sp.	Leaf-flower	Stems, leaves and flowers	2.40	Van der Ent et al., 2015
Phyllanthaceae	*Phyllanthus* sp. *nov. “*serinsim”	Phyllanthus	Leaves	158.00	Van der Ent et al., 2015
Phyllanthaceae	*Phyllanthus urinaria* L.	Chamber bitter	Leaves	12.00	Van der Ent et al., 2015
Pinaceae	*Picea abies* (L.) H.Karst.	Spruce	Needles	0.07	Reimann et al., 2001
Pinaceae	*Pinus sylvestris* L.	Pine	Needles	0.07	Reimann et al., 2001
Piperaceae	*Piper officinarum* C.DC.	Piper	Leaves	13.00	Van der Ent and Reeves, 2015
Poaceae	*Cymbopogan flexuosus* (Nees ex Steud.) Will.Watson	Lemongrass	Whole plants	0.30	Rajakaruna and Bohm, 2002
Poaceae	*Imperata cylindrica* (L.) Raeusch.	Alang grass	Leaves	0.03	Van Tran and Teherani, 1989
Poaceae	*Oryza sativa* L.	Rice	Seeds	0.04	Van Tran and Teherani, 1989
Poaceae	*Hordeum murinum* L.	Mouse Barley	Shoots	0.80	Koleli et al., 2015
Poaceae	*Aristida setacea* Retz.	Broom grass	Whole plants	14.60	Rajakaruna and Bohm, 2002
Polygonaceae	*Rumex obtusifolius* L.	Bitter dock	Shoots	0.80	Koleli et al., 2015
Rubiaceae	*Agrostemma cf. hameliifolium*	Corncockle	Leaves	16.00	Van der Ent and Reeves, 2015
Rubiaceae	*Canthium puberulum* Thwaites ex Hook.f.	Canthium	Stems, leaves and flowers	0.12	Rajakaruna and Bohm, 2002
Rubiaceae	*Canthium* sp.	Kidney-fruit Canthium	Stems, leaves and flowers	5.10	Rajakaruna and Bohm, 2002
Rubiaceae	*Morinda tinctoria* Roxb	Noni	Stems, leaves and flowers	0.70	Rajakaruna and Bohm, 2002
Rubiaceae	*Tarenna asiatica* (L.) Kuntze ex K.Schum	Tharana	Stems, leaves and flowers	1.10	Rajakaruna and Bohm, 2002
Rubiaceae	*Urophyllum* cf. *macrophyllum*	Urophyllum	Leaves	1.00	Van der Ent and Reeves, 2015
Salicaceae	*Salix* spp.	Willow	Leaves	1.76	Reimann et al., 2001
Solanaceae	*Physalis minima* L.	Cut-leaved ground-Cherry	Stems, leaves and flowers	3.90	Rajakaruna and Bohm, 2002
Taxodiaceae	*Taxodium distichum* (L.) Rich.	Bald cypress	leaves	4.60	McLeod and Ciravolo, 2007
Turneraceae	*Piriqueta duarteana* Urb.	Stripeseed	Leaves	11.00	Van der Ent and Reeves, 2015
Turneraceae	*Piriqueta* sp.	Stripeseed	Leaves	149.00	Van der Ent and Reeves, 2015
Turneraceae	*Turnera melochioides* A.St.-Hil. and Cambess.	Turnera	Leaves	143.00	Van der Ent and Reeves, 2015
Umbelliferae	*Conium maculatum* L.	Poison hemlock	Shoots	1.10	Koleli et al., 2015
Umbelliferae	*Sanicula europaea* L.	Sanicle, Wood sanicle	Shoots	5.40	Koleli et al., 2015
Violaceae	*Hybanthus enneaspermus* F.Muell.	Blue spade flower	Whole plants	17.00	Rajakaruna and Bohm, 2002

As discussed above, Co specific transporters have not been reported, and a schematic diagram for Co absorption and translocation is presented in Figure 2. After absorption by roots, Co is either sequestrated in the vacuole of root cells or transported to shoots. Co that is being transported to shoots is chelated with ligands. Co has little affinity with phytochelatins (Chen et al., 1997; Cheng et al., 2005), thus the ligands are not likely Co-S bonds. The study by Collins et al. (2010) reported that Co^2+^ was complexed with carboxylic acids, which were transported from roots to shoots in wheat or tomato plants. Other ligands are citrate or malate as well as non-proteinogenic amino acids, such as histidine and nicotianamine (Figure 2). Co has low mobility within the leaf tissue and is largely distributed in the vascular system of tomato and wheat leaves (Collins et al., 2010). Co transport from roots to shoots is well-controlled. Using radiolabeled ^57^Co, Page and Feller (2005) studied Co transport in wheat plants and found that 80% of ^57^Co remained in roots after 4 days of culture, and 50% was retained in the roots after 50 days; during which, some ^57^Co moved to the apical part of the main roots, suggesting that the loading of Co to the xylem is well-controlled, probably by FPN1 in wheat plants. In another study, Collins et al. (2010) reported that tomato and wheat plants grown in a nutrient solution containing 2.94 mg/L Co had 4,423 μg/kg and 9,319 μg/kg of Co in roots, respectively; but shoot concentrations of Co were 1,581 μg/kg and 395 μg/kg, respectively. This means that 35.7% of Co absorbed by tomato and 4.2% of Co absorbed wheat plants were transported from roots to shoots. Furthermore, for the 1,581 μg/kg Co in tomato shoots, 846 μg/kg was in the stem, 492 μg/kg in old leaves, only 243 μg/kg in young leaves, indicating that only 5.5% of absorbed Co is transported to actively growing shoots of tomato plants. These transport patterns are like those of titanium (Lyu et al., 2017) which are strictly controlled by plants. These findings imply that plants probably have unidentified transporters specifically for the transport of Co. Due to its toxicity at higher concentrations, the rigorous control of the transport and distribution would ensure that only an appropriate amount of Co could be transported to actively growing shoots. On the other hand, why was more Co transported to dicot tomato shoots than monocot wheat shoots? One explanation could be that different plants have different ligands for complexing Co, and Co complexed by ligands in tomato was more mobile than that in wheat. Another explanation could be that tomato plants need more Co to fulfill some unidentified roles in shoots. Further research is needed to verify these propositions.

To maintain ionic homeostasis in shoots, particularly in chloroplasts, plants develop mechanisms to mediate Co in chloroplasts. An ARG1 transporter, belonging to the ATP-binding cassette, was identified in rice (Li et al., 2020), which was able to modulate the levels of Co and Ni in chloroplasts to prevent excessive Co and Ni from competing with metal cofactors in chlorophyll and metal-binding proteins in photosynthesis (Figure 2).

### Plant Growth Improvement

Cobalt at low concentrations can also promote the growth of non-leguminous crops (Table 4). Co applied to a sandy soil at 1 mg/kg enhanced shoot and root dry weights of wheat by 33.7 and 35.8%, respectively compared with the control (Aery and Jagetiy, 2000), and the same Co rate applied to a sandy loam soil increased shoot and root dry weights of wheat by 27.9 and 39.6%, respectively, compared with the control. The yield and essential oil contents of parsley (*Petroselinum crispum*) increased considerably after the application of Co at 25 mg/kg soil (Helmy and Gad, 2002). Plant height, branch numbers, and fruit numbers as well as anthocyanin and flavonoids contents of *Hibiscus sabdariffa* significantly increased after application of Co at 20 and 40 mg/kg (Aziz et al., 2007). Application of 50, 100, 150, 200, and 250 mg/kg Co to corn plants showed that the root length, shoot height, and the number of cobs and seeds per plant increased when plants were applied with 50 mg/kg Co, but these parameters decreased with 100 mg/kg Co and above (Jaleel et al., 2009). Co applied at 10 mg/kg significantly enhanced the growth of two onion cultivars, bulb yields, bulb length, and bulb quality, such as nutrient and essential oil contents. Bulb diameter and bulb weights were much higher than the control treatment (Attia et al., 2014), but Co concentrations higher than 10 mg/kg significantly reduced the promotive effects.

**Table 4:** Effects of cobalt application on plant performance.

**Species (common name)**	**Co application**	**Effects on plants**	**References**
*Actinidia chinensis* Planch. cv. Hayward) (Kiwi)	Fruit was treated with 1 mM Co^2+^ solution	Inhibited ACC activity in ethylene biosynthesis	Hyodo and Fukasawa, 1985
*Adiantum raddianum* C. Presl (Delta maidenhair fern)	Cut green (frond) was treated with 1 mM Co(NO_3_)_2_ solution	Prolonged vase life of frond from 3 to 8.2 days	Fujino and Reid, 1983
*Arachis hypogaea* L. (Peanut)	Seeds were treated with Co(NO_3_)_2_ at 500 mg/kg seed and followed by two foliar sprays of cobalt nitrate at 500 mg/L before and after flowering	Significantly increased plant height, leaf number, pod yield, shelling percentage, harvest index, and total dry matter	Raj, 1987
*Arachis hypogaea* L. (Peanut)	CoSO_4_ was mixed with soil at 0.21 kg/ha	Resulted in 10% higher kernel yield compared with control (without Co application)	Basu and Bhadoria, 2008
*Arachis hypogaea* L. (Peanut)	Seedlings of groundnut at the third true leaf stage were irrigated once with CoSO_4_ at 2, 4, 6, and 8 mg/L, respectively	Increased plant height, number of branches and leaf number, leaf area index, root length, shoot and root biomass as well as pods numbers, pods weight, oil yield, total proteins, total carbohydrates, total soluble sugars, and total soluble solids	Gad, 2012a
*Argyranthemum* sp. (Argyranthemum)	Cut flowers preserved in a solution containing 2 mM Co	Increased flower longevity by more than 5 days compared with control (treated with distilled water)	Kazemi, 2012
*Avena sativ*a L. var. 'Condor' (Common oat)	Seeds were treated with 0.001% CoSO_4_ solution for 24 h, dried at room temperature for 3 days, then sown	Increased grain yields	Saric and Saciragic, 1969
*Beta vulgaris* L. (Red beet)	CoSO_4_ was mixed with soil at 2.5, 5.0, 7.5, 10.0 and 12.5 mg/kg, respectively	Increased plant growth, root yield, mineral elements as well as protein, carbohydrate, vitamin C, sucrose, and glucose contents	Gad and Kandil, 2009
*Cajanus cajan* (L.) Millsp. (Pigeon pea)	Seeds were treated with Co(NO_3_)_2_ at 500 mg/kg seed	Increased chlorophyll content, crop growth rate, relative growth rate, and net assimilation rate, resulting in increased plant height, number of branches, leaves, total dry matter, and yield	Raj, 1987
*Cariandrum sativum* L. (Coriander)	Irrigated in the form of CoSO_4_ 12.5 mg/L once	Increased coriander herb yield, mineral composition (except Fe), chemical constituents as well as essential oil components	Gad, 2012b
*Cicer arietinum* L. cv GG2 (Chickpea)	Chickpea seedlings at the three-leaf stage were fertigated with CoCl_2_ at 100 g/ha	Increased protein content and yield by 5.08 and 22.36%, respectively	Rod et al., 2019
*Cucumis sativus* L. cv. (Cucumber)	Plants were treated with Co(NO_3_)_2_ solutions ranging from 1 to 500 ?M	Promoted hypocotyl elongation	Grover and Purves, 1976
*Cucurbita pepo* cv. Eskandarany (summer squash)	Seeds in continuously aerated solutions of 0.25, 0.50, and 1.00 mg/L Co^2+^ for 48 h before sowing	Strongly increased plant growth, femaleness, and fruit yield compared with those of water- (control) or 0.5 mM AOA (aminooxyacetic acid)-soaked seed	Atta-Aly, 1998
*Allium cepa* L.) cv. Giza 6 Mohassan (Onion)	Co mixed with sand and petmoss in 10.0 mg/kg soil	Significantly promote nutrients and essential oils content along with bulb length, bulb diameter and weight	Attia et al., 2014
*Dianthus caryophyllus* L. cv. “Harlem” (Carnation)	Cut flowers were preserved in CoCl_2_ solutions at 50, 75, and 100 mg/L, respectively	Suppressed ethylene production and prolonged vase life	Jamali and Rahemi, 2011
*Gladiolus grandiflorus* Hort. cv. Borrega Roja (Gladiolus)	Plants were treated with solution containing 0.3 mM CoCl_2_	Increased stem and leaf N content, chlorophyll concentrations, leaf and stem dry weights, and improved stem absorption of water	Trejo-Téllez et al., 2014
*Glycine max* (L.) Merr. (Soybean)	Plants were grown in nutrient solutions containing 1 and 5 μg/L cobaltous chloride, inoculated with rhizobia in the absence of nitrogen	No N deficiency symptoms, and increased dry weight by 52% compared with the control treatments	Ahmed and Evans, 1959
*Glycine max* (L.) Merr. (Soybean)	Plants were grown in soil mixed with finely powdered (CoCl_2_) at the concentration of 50 mg/kg	Increased root and shoot length, leaf area, dry weight, yield, and yield components	Jayakumar et al., 2009
*Glycine max* (L.) Merr. (Soybean)	Seeds were sown in soil mixed with finely powdered (CoCl_2_) at 50 mg/kg	Increased yield parameters, leaf area, shoot length, total dry weight as well as total phenol percentage	Vijayarengan et al., 2009
*Hevea brasiliensis* (Willd. ex A.Juss.) Müll.Arg. (Rubber)	Plants were grown in Co free sand supplemented with 0.005 mg/kg Co	Increased plant height, stem diameter, and plant dry weight	Bolle-Jones and Mallikarjuneswara, 1957
*Hibiscus sabdariffa* L. (Roselle)	Seedlings irrigated once with Co at concentrations of 20 and 40 mg/L	Increased plant height, branch numbers, and fruit numbers as well as anthocyanin and flavonoids contents	Aziz et al., 2007
*Ipomoea batatas* L. (Sweet potato)	Seedlings were irrigated with CoSO_4_ once at concentrations of 5.0, 7.5, 10.0 mg/L	Increased growth and yield parameters, nutrient elements (except for Fe) and the chemical contents	Gad and Kandil, 2008
*Lilium* spp. cv. Star Gazer Lily	Cut flowers were preserved in a solution containing 0.1 mM Co and 4% sucrose with a pH of 3.5	Extended vase life	Mandujano-Piña et al., 2012
*Lilium* spp. cv. Prato (Lily)	Cut flowers were treated with 2 mM CoCl_2_	Increased vase life from seven to 9 days	Kazemi and Ameri, 2012
*Lilium* spp. cv. Star Fighter (Lily)	Cut flowers were preserved in solutions containing 0.1, 0.2 mM Co and 4% sucrose with a pH of 3.5	Extended the lifespan of flowers	Mandujano-Piña et al., 2012
*Lupinus angustifolius* cv. Uniharvest (Blue lupin)	Supplemented 0.9 mg CoSO_4_.7H_2_O to each pot containing 6 kg soil	Increased plant growth and N content	Robson et al., 1979
*Lycopersicon esculantum* Mill. (Tomato)	Ten seeds were sown in a pot containing 3 kg air-dried soil mixed with CoCl_2_ at 50 mg/kg, seedlings were thinned to 3	Increased the content of phosphorus, potassium, copper, iron, manganese, and zinc in plants	Jayakumar et al., 2013
*Lycopersicon esculentum* Mill. (Tomato)	Treated with simple solutions (1 mM CoCl_2_) plus wetting agent	Delayed gravitropic responses of treated plants	Wheeler and Salisbury, 1981
*Malus domestica* Borkh. (Apple)	Apple fruit was immersed in a solution containing 1 mM CoCl_2_ for 1 min	Enhanced activity of protein inhibitor of polygalacturonase (PIPG) and provided better conservation of apple fruit consistency during storage	Bulantseva et al., 2001
*Mangifera indica* L) cv. Langra (Mango)	Foliar spray with CoSO_4_ at 1,000 mg/L prior to flower bud differentiation in the first week of October	Reduced floral malformation by 65% and increased the fruit yield by 35%	Singh et al., 1994
*Matteuccia struthiopteris* (L.) Todaro	Supplemented with various concentrations Co^2+^ ranging from 0.1 to 1 mM	Inhibited IAA-induced ethylene production in sporophytes	Tittle, 1987
*Phaseolus aureus* Roxb. cv. T-44 (Mung bean)	Plants were treated with 50 μM Co in sand culture	Improved plant growth by increasing leaf, stem, and total dry weight compared with the controls	Tewari et al., 2002
*Phaseolus vulgaris* L. Cv. “Burpees Stringless” (Common bean)	Two cycles of pre-sowing soaking and drying treatments by a 1 mg/L of Co(NO_3_)_2_ solution	Increased yield and N content over untreated and distilled water-soaked seeds by 48 and 150%, respectively	Mohandas, 1985
*Pisum sativum* L. (Garden pea)	Seeds sowed in pot containing 10 kg soil mixed with CoSO_4_ at 8 mg/kg	Enhanced N_2_ fixation process, increased plant N content, and reduced inorganic and organic N fertilizer application by 75 and 33.3%, respectively	Gad, 2006
*Pisum sativum* L. (garden pea)	Pots filled with 10 kg soil with Co at 2 mg/kg	Increased grain yield by 48.4%	Singh et al., 2012
*Polianthes tuberosa* L. (Tuberose)	Flower stems were preserved in a solution containing 300 mg/L cobalt chloride	Extended the vase life and enhanced water uptake in cut tuberose flowers	Mehrafarin et al., 2021
*Pteridium aquilinum* (L.) Kuhn var. *latiusculum* (Desv.) Underw. ex Heller (Western bracken fern)	Stems of cut green were preserved in solutions containing 0.1 to 1.0 mM Co	Inhibited IAA-induced ethylene production and prolonged vase life	Tittle, 1987
*Ricinus communis* L. (Castor bean)	Plants treated with a 1 mM CoCl_2_ solution supplemented with a wetting agent	Delayed gravitropic responses of treated plants	Wheeler and Salisbury, 1981
*Rosa hybrida* “Samantha” (Rose)	Cut flowers were preserved in solutions containing 0.5, 1.0, 1.5, and 2.0 mM CoCl_2_, respectively	Increased leaf diffusive resistance, inhibited xylem blockage, maintained water flow and uptake, and increased the vase life	Reddy, 1988
*Rosa* hybrida “Samantha” (Rose)	Cut flowers were preserved in solutions containing 0.5, 1.0, 1.5, and 2.0 mM Co(NO_3_)_2_, respectively	Highly delayed or prevented the development of bent-neck and increased water uptake of cut flower	Murr et al., 1979
*Rosa* spp.cv. Red one (Rose)	Cut flowers treated with 100 and 200 mg/L Co solutions	Inhibited vascular blockage in the stem of rose and maintained a high-water flow rate, leading to significantly water uptake by cut flowers	Aslmoshtaghi, 2014
*Triticum aestivum* L. (Wheat)	Seeds were sowed in polythene-lined pots containing 4 kg of soil mixed with 1 mg/kg CoSO_4_	Enhanced plant growth after 45 days of application	Aery and Jagetiy, 2000
*Vicia faba* L. (Fava bean)	Seedlings at six-leaf stage were planted in pot containing soil mixed cobalt at 20 and 40 mg/kg, respectively	Improved photosynthesis and plant growth	Wang et al., 2015
*Vigna anguiculata* subsp. *alba* (G. Don) Pasquet (Cowpea)	Seedlings were applied with Co at 4, 6, and 8 mg/kg	Enhanced plant growth and yield and induced nodulation	Gad and Hassan, 2013
*Xanthium strumarium* L. (Cocklebur)	Plants treated with a 1 mM CoCl_2_ solution supplemented with a wetting agent	Delayed gravitropic responses of treated plants	Wheeler and Salisbury, 1981
*Zea mays* L. (Maize)	Seeds sowed in pots containing 13 kg soil mixed with Co at 50 mg/kg	Increased seedling growth, photosynthetic pigments, and leaf chlorophyll contents	Jaleel et al., 2009

Explanations for the improved growth of non-leguminous plants vary but can be summarized as follows: (1) enhanced tolerance to abiotic stresses, (2) activation of antioxidative enzymes, (3) substitution of active metals, and (4) hormesis. Application of Co has been reported to alleviate drought, salt, heavy metal stresses, thus plant growth is not adversely affected. Co has been reported to suppress plant uptake of cadmium (Chmielowska-Bak et al., 2014). Co application increased free proline accumulation, which counteracted the salt stress. In general, abiotic stresses often cause plant imbalance between production and accumulation of reactive oxygen species (ROS), including superoxide anion (${\text{O}}_{2}^{-}$), hydroxyl radical (OH^−^), and hydrogen peroxide (H_2_O_2_) (Sachdev et al., 2021). ROS can activate the antioxidant system of the plant, thus minimizing the damages (Tewari et al., 2002; Choudhury et al., 2017). The antioxidant system includes enzymatic antioxidants: ascorbate peroxidase, catalase, dehydroascorbate reductase, general peroxidases, glutathione reductase, monodehydroascorbate reductase, and superoxide dismutase as well as non-enzymatic antioxidants, mainly ascorbic acid, α-tocopherol, carotenoids, reduced glutathione, plastoquinone/ubiquinone, and flavonoids (García-Caparrós et al., 2020). The action of the antioxidant system could be the first line of defense against the adverse effects. Therefore, it is not surprising to notice increased activities of ascorbate peroxidase, catalase, peroxidase, and superoxide dismutase (Hasanuzzaman et al., 2020). Co applied at appropriate concentrations can activate antioxidative enzymes, thus reducing ROS-caused damage. As discussed previously, Co may substitute other nutrient elements when such nutrients have limited availability. Baxter et al. (2008) showed that when *Arabidopsis* plants are grown under a low Fe concentration, the shoot concentration of Co increased, which was concomitant with the increased expression of Fe transporter IRT1. Additionally, Co contribution to hormesis has been proposed (Shahid et al., 2020). Due to the limited research on Co to date, these explanations may not be on target and incomplete. Our proposition is that the application of the appropriate amount of Co may stimulate rhizosphere bacteria (either symbiotic, endophytic, or associated ones) to fix N_2_, increase soil N, and enhance plant growth. Concomitantly, Co enzymes may be triggered to conduct proper biochemical and physiological activities, such as carbonate dehydratase may enhance photosynthesis and Co-peroxidase may activate the enzymatic antioxidant system. As a result, healthy growing plants would take up more nutrients from the soil and improve their growth and overall stress tolerance.

### Other Performance Enhancement

Cobalt has been shown to have other beneficial effects on plants. Co as a component of preservative solutions can improve the postharvest quality of floriculture crops by prolonging the vase life of cut flowers. Cut fronds of Delta maidenhair fern (*Adiantum raddianum*) placed in deionized water became wilted in just 3 days because of the vascular blockage at the basal end of the petiole. The wilting, however, could be delayed for up to 8 days by adding 1 mM Co as Co(NO_3_)_2_ to the water (Fujino and Reid, 1983). The delay of senescence is attributed to the antibacterial activity of Co (Van Doorn et al., 1991). Co addition to preservative solutions increased leaf diffusive resistance, reduced xylem blockage, sustained water flow and uptake, and prolonged vase life of cut flowers of *Rosa hybrida* “Samantha”. Reddy (1988) suggested that partial closure of stomata by Co was responsible for reducing the water loss/water uptake ratio, and thereby maintaining a higher water potential in the cut roses. Co was also reported to slow the senescence process in harvested lettuce (Tosh et al., 1979). Co^3+^ has been reported to form Co-complexes, which have antiviral activities (Chang et al., 2010). In addition to antibacterial and antiviral activities, Co shows inhibitory activity to 1-aminocyclopropane-1-carboxylic acid (ACC) oxidase. Ethylene is synthesized from amino acid methionine by two key enzymes, ACC synthase, and ACC oxidase. Co can block the conversion of ACC to ethylene by inhibiting ACC oxidase activity in the ethylene biosynthesis pathway (Lau and Yang, 1976; Serek et al., 2006), thus increasing the vase life of cut flowers.

## Cobalt Deficiency Occurs in Plants

Cobalt deficiency does occur in plants. Its deficiency symptoms include leaf chlorosis and necrosis, growth retardation, and reduced crop yield, resembling N-deficiency in plants (Liu, 1998). Co deficient legumes have reduced plant size, smaller and pale-yellow leaves, and smaller pods compared with non-deficiency plants. Root growth is also affected by exhibiting an overall reduction of root volume and root lengths. Nodule size and numbers are less abundant than the plants without Co deficiency. Co deficiency causes reduced synthesis of methionine, thus limiting protein synthesis and contributing to the smaller-sized bacteroids (Marschner, 2011). Sweet lupin is particularly sensitive to Co deficiency (Robson et al., 1979). In field-grown lupins, Co deficiency reduced bacteroid number per gram of nodule (Chatel et al., 1978) and affected nodule development and function at different levels (Dilworth et al., 1979). Co deficiency in legumes can be assessed by analysis of Co contents in shoots. In general, deficient symptom appears when shoot Co falls in a range from 0.04 (Ozanne et al., 1963) to 0.02 mg/kg based on dry weight (Robson et al., 1979). To correct Co deficiency in leguminous crops, application of Co in a range of 1.8 to 145.6 g per hectare was reported (Havlin et al., 2013).

Cobalt deficiency also occurs in non-leguminous plants. Co deficiency causes growth retardation in rubber trees and tomato plants (Wilson and Nicholas, 1967). Symptoms of Co deficiency in corn and wheat showed leaf chlorosis and reduced growth (Wilson and Nicholas, 1967). Low leaves may become necrotic, root systems are reduced with decreased number of N_2_ fixing bacteria. Grasses with low contents of Co can result in Co deficiency of sheep and cattle. For countries, like South Australia, Sierra Leone, Malta, New Zealand, and Finland, where soils have low Co contents (Sillanpaa and Jansson, 1992), application of Co could improve forage grass growth and enrich tissue Co content. Thus, the feeding of ruminants with healthy grass can reduce Co deficiency (Lee, 1951; Dewey et al., 1958). Due to low Co concentrations in plants, Co deficiency in grazing animals may occur, which can be corrected by mixing Co salts with fertilizers or sand carriers to broadcast it over grazed pastures.

## Cobalt Toxicity in Plants

Cobalt at high concentrations causes cytotoxicity and phytotoxicity in plants, which is similar to Cu, Ni, and Zn. Cytotoxicity is the inhibition of mitosis and damage of chromosomes, and disruption of the endoplasmic reticulum of root tip cells (Rauser, 1981; Smith and Carson, 1981; Akeel and Jahan, 2020). Phytotoxicity varies depending on plant species and the concentration of Co in plant organs. Leguminous plants generally exhibit chlorosis or pale-white color on young leaves, and tomatoes show either interveinal chlorosis or diffused chlorosis on young leaves (Akeel and Jahan, 2020).

Cobalt toxicity to plants is uncommon in natural soils, but it happens when plants grow in Co contaminated soils. Soil contamination by Co is mainly from mining and smelting activities, disposal of sewage sludge, and the use of chemical fertilizers (Hamilton, 1994). As discussed above, plants can control Co absorption, transport, and distribution. However, when Co in contaminated soils becomes highly available, Co may gain a competitive advantage over Fe, resulting in more Co being absorbed than Fe through IRT1. With increasing concentrations of Co inside cells, FPN2 may not be able to effectively sequester Co into the vacuole, resulting in more Co to transport from roots to shoots. Li et al. (2020) showed that Co concentrations in shoots of barley, oilseed rape (*Brassica napus*), and tomato were linearly correlated with the soil solution Co. As a result, excessive Co in shoots may initially cause oxidative stress, resulting in increased anti-oxidative enzyme activities (Tewari et al., 2002). As the stress progresses, Co may compete with Fe or Mg in the chloroplast by decreasing chlorophyll content (Lwalaba et al., 2017), which causes Fe deficiency with newly growing leaves to be yellowish in color. As reported by Sree et al. (2015), Co is able to inhibit the activity of enzymes involved in the biosynthesis of chlorophyll intermediates, like 5-aminolevulinic acid and protoporphyrin, which will reduce net photosynthetic activities. Co also adversely affects the translocation of P, S, Cu, Mn, and Zn from roots to shoots (Chatterjee and Chatterjee, 2000). All these factors, acting together, can result in phytotoxicity and significantly reduce plant growth.

Different plants show different abilities to tolerate Co. Oat (*Avena sativa*) plants were adversely affected when grown in a soil solution containing 0.14 mg/L Co (Anderson et al., 1973). Rice (*Oryza sativa*) plants would develop toxic symptoms when grown in soils with Co ranging from 25 and 50 mg/kg (Kitagishi and Yamane, 1981). The contents of Co could be used for predicting the development of toxicity (Akeel and Jahan, 2020). Toxic symptoms occurred in bush beans when tissue Co contents ranged from 43 to 142 mg/kg (Wallace et al., 1977); similarly, 6 mg/kg in barley seedlings (Davis et al., 1978), and 19 to 32 mg/kg in Sudan grass (Gough et al., 1979). In general, tissue Co contents between 30 and 40 mg/kg are considered critical levels for the potential development of Co toxicity (Macnicol and Beckett, 1985). However, due to evolutionary adaptation, Co hyperaccumulators do not develop toxic symptoms at this concentration level. Co contents in leaves of *Rinorea* cf. *bengalensis* can be 1,200 mg/kg (Paul et al., 2020), and *Glochidion* cf. *sericeum* can accumulate 1,500 mg/kg Co (Van der Ent et al., 2018). Co hyperaccumulators are not the focus of this article. The reader is referred to publications by Brooks (1977), Brooks et al. (1977, 1980), Baker (1981, 1987), Lange et al. (2017), and Yamaguchi et al. (2019) for more information.

## Conclusions and Future Perspectives

Cobalt in soils ranges from 15 to 25 mg/kg, wherein plant roots can absorb Co from soils and transport absorbed Co from roots to shoots in a controlled manner. Co concentrations in shoots vary with plant species but are comparable to those of essential elements of Cu, Ni, and Zn. Co was well-documented as a constituent of cobalamin, which is required by symbiotic, endophytic, and associated bacteria in the fixation of N_2_. Biological N fixation contributed significantly to the production of economically important crops, including beans, soybeans, rice, corn, barley, wheat, and sugarcane. The current view of plant-microbe association as a phytomicrobiome resulted from millions of years of co-evolution. The coevolution between plants and N_2_ fixing bacteria should remind us of the critical role Co plays and its potential essentiality to plant growth and development. Additionally, plants must have Co enzymes or proteins that are specifically responsible for Co metabolism. Due to its similar properties to other transition elements, its biological roles in plants have been largely ignored and simply attributed to its ability to substitute for those elements.

Further research is warranted to (1) identify specific roles of Co plays in diazotrophs, with an emphasis on endophytic and associated bacteria, (2) ascertain Co-containing enzymes and proteins that are implicated in metabolisms of both lower and higher plants, (3) determine the interactions of Co with other transition metals in the regulation of enzymatic activities, (4) recognize Co as an essential micronutrient for plant growth, and (5) develop nutrient management programs by incorporating a group of particular N fixing bacteria with the appropriate amount of Co as plant-specific fertilizers for improving crop production. With the advance in omics, these tasks should be accomplished in the near future. The recognition of Co as an essential micronutrient would enrich our understanding of plant mineral nutrition and enhance crop productivity.

## Author Contributions

XH, XW, and JC wrote the manuscript. JL prepared figures. All authors contributed to the acquisition and interpretation of available literature and the conception of the work, revised the manuscript, and approved this final version.

## Funding

This study was supported in part by the Students Innovation and Entrepreneurship Training Program at Zhongkai University of Agriculture and Engineering with grant number 202111347007 and the General Project of the Natural Science Foundation of Fujian Province with grant number 2020J01867.

## Conflict of Interest

The authors declare that the research was conducted in the absence of any commercial or financial relationships that could be construed as a potential conflict of interest.

## Publisher's Note

All claims expressed in this article are solely those of the authors and do not necessarily represent those of their affiliated organizations, or those of the publisher, the editors and the reviewers. Any product that may be evaluated in this article, or claim that may be made by its manufacturer, is not guaranteed or endorsed by the publisher.
